# miR-4429 inhibits ccRCC proliferation, migration, and invasion by directly targeting CD274

**DOI:** 10.1007/s12672-024-01055-4

**Published:** 2024-05-27

**Authors:** GuangYi Hong, YiKun Wu, ShiYu Huang, Yang Hu, Ying Zhang, CiCi Guo, Hua Shi, ShuXiong Xu

**Affiliations:** 1 Guizhou University Medicine College, Guiyang, 550025 Guizhou China; 2 Department of Urology, Tongren City People’s Hospital, Tongren, Guizhou China; 3 Department of Urology, Guizhou Provincial People’s Hospital, No. 83, East Zhongshan Road, Guiyang, Guizhou China

**Keywords:** Clear cell renal cell carcinoma, miR-4429, CD274, PD-L1, PI3K/AKT

## Abstract

Clear cell renal cell carcinoma (ccRCC) is one of the most aggressive urological malignancies and a highly immunogenic cancer. Yet, its pathogenesis is still not fully understood. This study analyzed the role of the miR-320 family in ccRCC using bioinformatics algorithms and a series of in vitro experiments. miR-4429 was found to be significantly down-regulated in ccRCC tissues and cell lines, while overexpression of miR-4429 significantly inhibited renal cancer cell proliferation, migration, and invasion in vitro. In addition, the UALCAN database, immunohistochemistry, and protein blotting results showed that CD274 expression was up-regulated in ccRCC tissues and correlated with higher histologic grading. Dual luciferase assay indicated that CD274 was a direct target of miR-4429. Overexpression of miR-4429 in 786-O, Caki-2 cells significantly inhibited CD274 expression. KEGG results indicated that the potential target function of miR-4429 was associated with the PI3K/AKT signaling pathway, and protein blotting verified the results. In summary, this data shows that miR-4429 targets CD274 and inhibits ccRCC proliferation, migration, and invasion by regulating PI3K/AKT signaling, thus potentially providing a promising therapeutic target and prognostic biomarker for renal cell carcinoma patients.

**Graphical Abstract:**

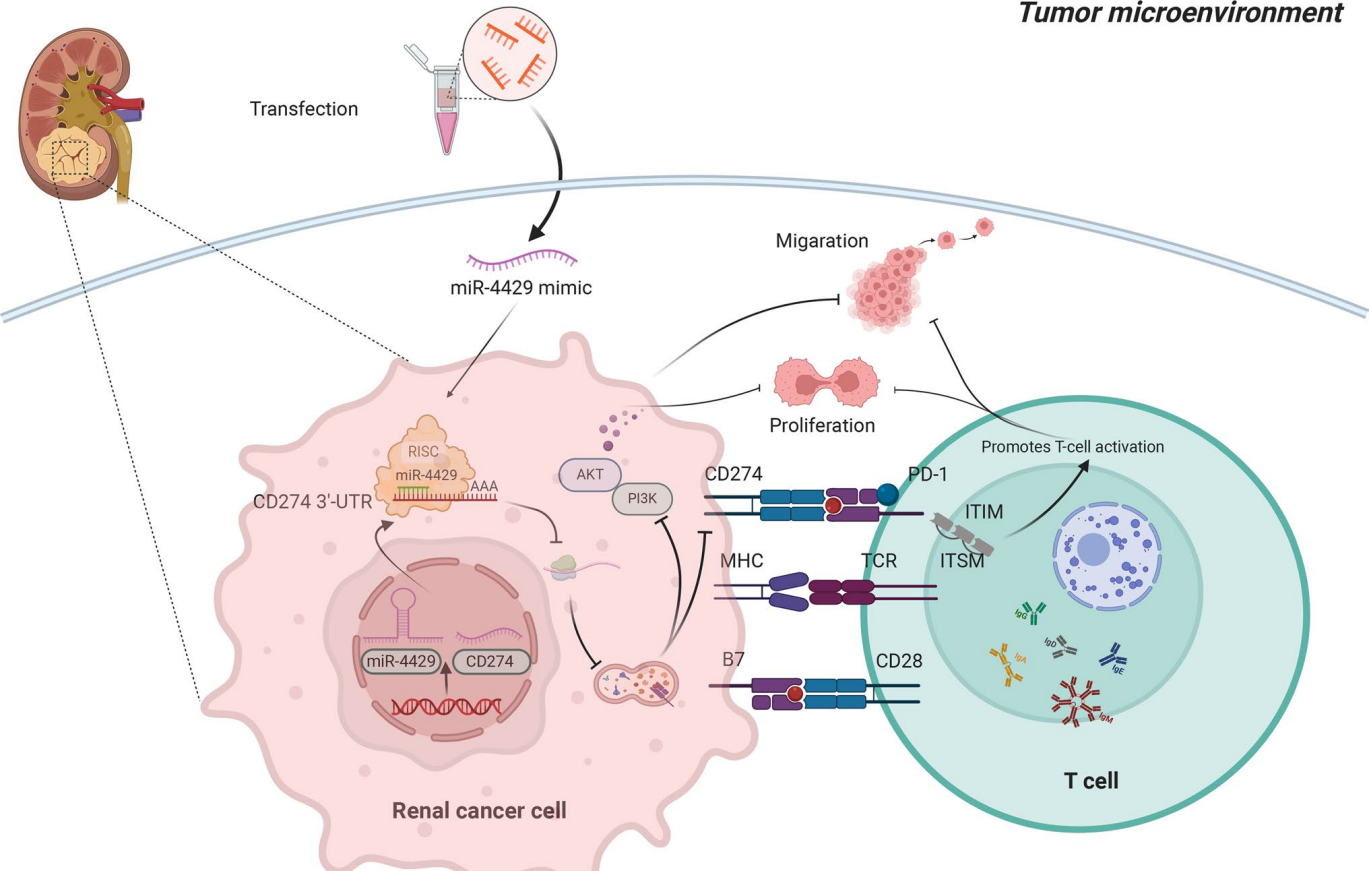

**Supplementary Information:**

The online version contains supplementary material available at 10.1007/s12672-024-01055-4.

## Introduction

Renal cell carcinoma (RCC) ranks as the third most prevalent urinary tract neoplasm, constituting about 90% of renal malignancies and 4% of all malignant tumours in the adult population [1]. Clear cell RCC (ccRCC), the most common and aggressive RCC subtype, represents 70–75% of RCC cases [2]. Owing to its elusive early clinical manifestations, more than 30% of ccRCC patients manifest metastases at their initial diagnosis, thereby forgoing surgical options. Additionally, roughly 40% of patients who underwent primary ccRCC resection still experienced cancer recurrence and metastasis, resulting in an overall 5-year survival rate less than 10% [3, 4]. Consequently, exploring the molecular underpinnings of ccRCC development and identifying potential targets for ccRCC assume paramount importance in enhancing ccRCC treatment.

Micro RNAs (miRNAs) are a class of small non-coding RNAs, consisting of approximately 22 nucleotides, that are indispensable factors in the regulation of tumorigenesis and metastasis [5]. miRNAs function as either tumour suppressors or oncomiRNAs by binding to the 3′-untranslated region (3′-UTR) of target genes, thereby downregulating the transcription of associated genes [6]. Extensive reports indicate that miR-320 family members, including miR-320a, miR-320b, miR-320c, miR-320d, and miR-4429 are downregulated in various malignancies and have been linked to tumour aggressiveness and an unfavourable prognosis [7–11]. Nevertheless, there is a paucity of studies on the involvement of miR-320 family members in the onset and progression of ccRCC.

This study undertook an analysis of the miR-320 family's role in ccRCC using bioinformatic algorithms and a series of in vitro experiments. These findings substantiate the notion that miR-4429 targets CD274 to curtail ccRCC proliferation, migration, and invasion by modulating the PI3K/AKT signalling pathway.

## Materials and methods

### Bioinformatics analysis

The Cancer Genome Atlas (TCGA, https://www.cancer.gov/ccg/research/genome-sequencing/tcga) served as the primary source for miR-320 family data and associated information. Additionally, the miRNA target prediction databases, miRDB (http://www.mirdb.org/) and TargetScanv7.1 (http://www.targetscan.org/), were utilised to forecast potential targets of miR-4429. All putative targets were analyzed with functional enrichment analysis in KEGG (http://www.genome.jp). Furthermore, the UALCAN database (https://ualcan.path.uab.edu/analysis.html) was employed to assess the differential expression and histological correlations of CD274 in ccRCC. Timer2.0 (http://timer.comp-genomics.org/), an online resource, was harnessed to investigate associations between PD-1/CD274 and the links between CD274 and indicators of immune infiltration.

### Clinical samples

A total of 30 paired tumour and normal tissues were obtained from ccRCC patients between January 2022 and June 2023 at the Department of Urology, Guizhou Provincial People's Hospital. Surgical specimens were promptly subjected to flash freezing in liquid nitrogen for preservation. Clinical information pertaining to patients, encompassing gender, age, TNM classification, tumour size, and related variables, was meticulously recorded. All patients received a histological diagnosis of ccRCC and had not undergone radiotherapy or chemotherapy prior to surgery.

The present study was approved by the Ethics Committee of Guizhou Provincial People’s Hospital, and all participants provided written informed consent. A summary of patient information can be found in Table 1.

**Table 1:** Clinical information on patients included

Characteristics	n (%)
Gender	
Female	12 (40%)
Male	18 (60%)
Age	
< 60	21 (70%)
≥ 60	9 (30%)
Fuhrman grade^a^	
G1	4 (13.3%)
G2	9 (30%)
G3	15 (50%)
G4	2 (6.6%)
pT category^b^	
pT1	7 (23.3%)
pT2	5 (16.6%)
pT3	14 (46.6%)
pT4	4 (13.3%)
Metastasis	
No metastasis	22 (73.3%)
Metastasis	8 (26.6%)

^a^Based on Fuhrman grade classification

^b^According to pTNM classification

### Cell culture

We procured human ccRCC cell lines, namely ACHN, 786-O, Caki-1, Caki-2, and human renal cortical proximal tubular epithelial cells, HK-2, from Pronox (Wuhan, China). Specifically, 786-O cells were cultured in RPMI 1640 medium, ACHN and HK-2 in MEM medium, and Caki-1 and Caki-2 in McCoy’s 5A medium. Each complete medium was supplemented with 100 U/mL penicillin, 100 U/mL streptomycin, and 10% fetal bovine serum (FBS). These cells were maintained in a humidified environment comprising 5% CO_2_ and 95% air at 37 °C.

### Cell transfection

We collected 786-O and Caki-2 cells during the logarithmic growth phase, enumerating them to 2 × 10^6^ cells/mL and inoculated into six-well plates. Transfection was performed when the cells reached 50% to 60% confluence. The miR-4429 mimic, mIR-4429 inhibitor, and miR-NC negative control were designed and synthesized by Biotech Bioengineering (Shanghai, China), 50 nM miRNA was transfected with Lipofectamine 2000 (Invitrogen; Thermo Fisher Scientific, Inc.) into 786-O and Caki-2 cell lines according to the manufacturer's protocol. Cells were collected 48 h after transfection to carry out the following experiments.

### Quantitative real-time PCR (qRT-PCR)

Total RNA was extracted from frozen samples derived from 786-O and Caki-2 cell lines that had been transfected with miR-4429 mimics, inhibitors, and negative controls, along with samples from ccRCC surgery, utilising the Total RNA Extraction Kit (TaKaRa, Japan). For miRNA extraction, we employed the miRNA Extraction Kit (Tiangen, China). Following the kit instructions, cDNA was generated through miRNA reverse transcription, and the concentration and purity of the synthesised cDNA were evaluated with Nanodrop One. To ascertain the expression of miR-320 family members, we conducted qRT-PCR using the SYBR Green kit (Applied Biosystems, Foster City, USA) on a Mastercycler RT-PCR system (Eppendorf, Germany). Relative expression levels were determined via the 2^−ΔΔCt^ method. The primer sequences are detailed in Table 2.

**Table 2:** Sequences of primers for qRT-PCR and transfection

Name		
miR-4429-3p	Forward	5′-GGCCAGGCAGRCTGAGTTG-3′
	Reverse	5′-GGGAGAAAAGCTGGGCTGAG-3′
miR-4429-3p mimic	Sense	5′-AAAAGCUGGGCUGAGAGGCG-3′
	Antisence	5′-CCUCUCAGCCCAGCUUUUUU-3′
miR-4429-3p mimic NC	Sence	5′-UUGUACUACACAAAAGUACUG-3′
	Antisence	5′-GUACUUUUGUGUAGUACAAUU-3′
miR-4429-3p inhibitor	Sence	5′-CGCCUCUCAGCCCAGCUUUU-3′
miR-4429-3p inhibitor NC	Sence	5′-CAGUACUUUUGUGUAGUACAA-3′
CD274	Forward	5′-CTTGAACCCTTGAATGCC-3′
	Reverse	5′-CTGAATCTCGAAACCTCCA-3′
GAPDH	Forward	5′-CAGGAGGCATTGCTGATGAT-3′
	Reverse	5′-GAAGGCTGGGGCTCATTT-3′
U6	Forward	5′-CTCGCTTCGGCAGCACA-3′
	Reverse	5′-AACGCTT CACGAATTTGCGT-3′

### Cell proliferation assay

We employed the Cell Counting Kit-8 (CCK-8) (Dojindo, Japan) to evaluate in vitro cell proliferation. 786-O and Caki-2 cells, which had been transfected with miR-4429-mimic, miR-4429-inhibitor, and miR-NC, were gathered and seeded into 96-well plates at a density of 5 × 10^3^ cells per well for time points of 0 h, 24 h, 48 h, 72 h, and 96 h. At each designated time point, 10 µL of sterile CCK-8 solution was introduced into each well, followed by an incubation period of 1–2 h at 37 °C. The absorbance at 450 nm was quantified using a microplate reader (SpectraMax Plus 384, United States of America).

### Colony formation assay

Transfected cells were seeded into six-well plates at a density of 500 cells per well and cultured for 15 days in a medium containing 10% FBS. Subsequently, they were washed three times with PBS at room temperature, fixed with 4% paraformaldehyde (PFA) for 15 min, stained with 1% crystal violet for 20 min, and then subjected to photographic documentation and counting utilising Image J.

### Wound healing assay

Cells were seeded in a six-well petri dish, once the cell monolayer reached approximately 90% confluence, it was manually disrupted by scraping with the tip of a P-20 microtube. Cell migratory capacity was assessed through the monitoring and measurement of wound healing.

### Cell migration and invasion assays

We employed Transwell chambers (Corning Costar Corp., Cambridge, MA, USA) with 8 μm pores, placed in 24-well culture plates. For the invasion assay, matrix gel (BD Bioscience, USA) stored at − 20 °C was allowed to dissolve overnight at 4 °C. It was subsequently diluted in a 1:8 ratio with FBS-free cell-affiliated medium, and 100 µL of the gel mixture was used to coat the upper chamber evenly, followed by an incubation period at 37 °C for 2 h. 786-O and Caki-2 cells, transfected for 48 h, were diluted with RPMI 1640 and McCoy's 5A medium without FBS, to a concentration of 5 × 10^5^ cells per 200 µL, and seeded into the upper chamber. The lower chamber was supplemented with RPMI 1640 and McCoy's 5A medium containing 10% FBS. After 48 h of incubation, cells were fixed with 4% PFA for 15 min at room temperature and then stained with 1% crystal violet for 20 min. Subsequently, five different field of view statistics were taken at random.

### Western blot

To prepare cell lysates, cells were collected at 70–80% confluence 48 h post-transfection. Protein concentrations were determined, and a loading buffer was prepared according to the Solabank BCA Protein Concentration Assay Kit (Beijing, China). The prepared loading buffer was applied to individual lanes on a 4–15% gradient polyacrylamide gel (Mini-PROTEAN TGX™, Bio-Rad). Blotting was executed using a gel transfer system (Invitrogen) to transfer proteins onto PVDF membranes, which were then immersed in TBST (TBS containing 5% skim milk powder and 0.1% Tween) for 2 h. Following this, the blotted membranes were incubated with primary antibodies at a concentration of 1:1000 overnight at 4 °C. After washing the membrane three times, it was incubated with rabbit-specific secondary antibody (1:3000) for 2 h at room temperature. To develop the blotted membrane, the membrane was washed thrice with the ELC kit (New Cell & Molecular Biotech Co., Ltd. SuZhou, China), and the grayscale values of the target bands were assessed utilising Image J. GAPDH expression level was employed to normalise the target proteins. All antibodies were sourced from Abcam, including CD274 (ab205921), PI3K (ab302958), p-PI3K (#4228), p-AKT (ab8805), AKT (ab8805), and GAPDH (ab8245).

### Dual-luciferase reporter assay

We amplified the 3′-UTR of CD274, which carried the putative binding site for miR-4429, and then cloned it into the pmirGLO vector. To eliminate complementarity with miR-4429, the miR-4429 complementary site within the CD274 3′-UTR, with the sequence 5′-CAGCUUU-3′, was individually mutated. 293T cells were seeded into 96-well plates at a density of 4000 cells per well and transfected with luciferase reporter vectors containing either the wild-type (WT) or mutated (MUT) CD274 3′-UTR (constructed by Ribobio), along with miR-4429 mimics or a negative control (NC). After 48 h of transfection, cells were harvested, and the Dual-Luciferase® Reporter Assay System was utilised to measure luciferase activity. Normalised luciferase activity was determined as the ratio of luciferase activity to Renilla luciferase activity.

### Immunohistochemical staining

For immunohistochemistry, the hydrated sections were treated with an antigen repair solution (Shanghai Shumba Biotech; Shanghai, China). Subsequently, the slides were soaked in 3% H2O2 for 20 min and then incubated with Primary antibody (CD274, 1:150) was incubated at 4 °C for 12 h. The following day, the slides were rinsed and incubated with rabbit secondary antibody at 15 °C for 90 min and then transferred to 37 °C for 30 min. Separate staining with 3,3′-diaminobenzidine (DAB) and hematoxylin was conducted. The slides were then assessed and captured using an Olympus BX53 fluorescence microscope (Tokyo, Japan).

### Statistical analysis

We conducted statistical analyses utilising SPSS 19.0 (IBM SPSS, Chicago, IL) and GraphPad Prism 6.0 (IBM, Armonk, NY, USA). Paired and unpaired samples were analyzed for statistical significance using t-tests. For samples with more than two groups, analysis of variance (ANOVA) was used. Correlations between gene expression levels were analyzed using Spearman's correlation analysis. All experiments were repeated at least three times. Statistical significance was set at P values < 0.05.

## Results

### MiR-4429 is significantly down-regulated in ccRCC tissues

To investigate the potential association of miR-320 family members with the progression of ccRCC, we conducted a comprehensive analysis of their expression patterns utilising the TCGA database and bioinformatics algorithms. The analysis was based on three dimensions, including differential expression, prognosis, and metastasis (relative to normal tissue) in ccRCC. In terms of prognostic stratification, we segregated patients into two groups: those with less than one year of survival and those with over five years of survival. In the context of metastasis, ccRCC patients were categorised into metastatic (M1) and non-metastatic (M0) groups.

**Fig. 1:**
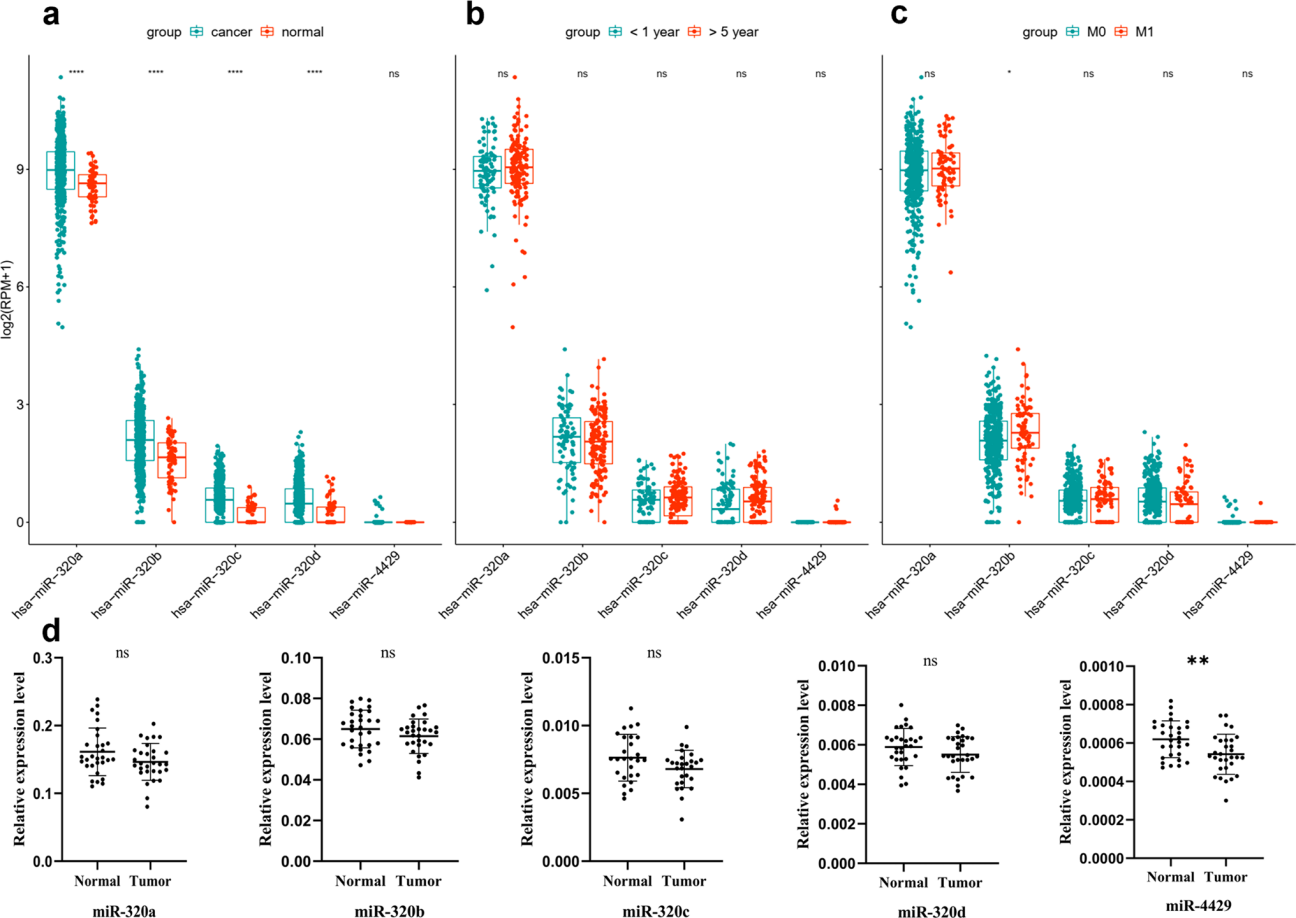
MiR-4429 was significantly downregulated in ccRCC tissues **a–c** MiR-320a, miR-320b, miR-320c, and miR-320d expression were up-regulated in the **a** differentially expressed group compared to normal tissues. miR-320 family members were not significantly different in the **b** prognosis group, and only miR-320b was significantly different in the **c** metastasis group, **d** The expression levels of miR-320 family members were detected in the tissues of 30 ccRCC patients, and miR-4429 expression was significantly down-regulated. *P < 0.05, **P < 0.01, ***P < 0.001, ****P < *0.0001*

Differential expression analysis revealed up-regulation of miR-320 family members, including miR-320a, miR-320b, miR-320c, and miR-320d, as depicted in Fig. 1a. However, when considering prognosis (Fig. 1b), there was no significant differences. In contrast, miR-320b exhibited significant variation in the context of the metastatic group, as shown in Fig. 1c.

To further validate the expression patterns of miR-320 family members in ccRCC patients, we conducted RT-qPCR to assess their levels in 30 ccRCC tumour samples and compared with control tissues. The outcomes revealed a noteworthy down-regulation of miR-4429 in ccRCC tissues. However, the expression levels of miR-320a, miR-320b, miR-320c, and miR-320d did not exhibit statistically significant differences, as illustrated in Fig. 1d. This finding suggests that miR-4429, a member of the miR-320 family, may serve as an independent biomarker in the context of ccRCC.

### Overexpression of miR-4429 inhibits proliferation, migration, and invasion of 786-O and Caki-2 cells in vitro

In order to elucidate the involvement of miR-320 family members in the proliferation and metastasis of ccRCC, we employed miR-4429, a representative of this family, and utilised RT-qPCR to assess its expression levels in various cell lines including HK-2, ACHN, 786-O, Caki-1, and Caki-2. Subsequently, we transfected 786-O and Caki-2 cells with mimic, inhibitor, and negative control to gauge transfection efficiency. The biological function of miR-4429 was rigorously examined through a battery of assays, including cell viability assay performed with CCK-8, clone formation assay, wound healing assay, and Transwell assay. Predictive target analysis of miR-4429 was conducted using miRDB and TargetScanv7.1, and all putative targets underwent functional enrichment analysis employing the KEGG database.

**Fig. 2:**
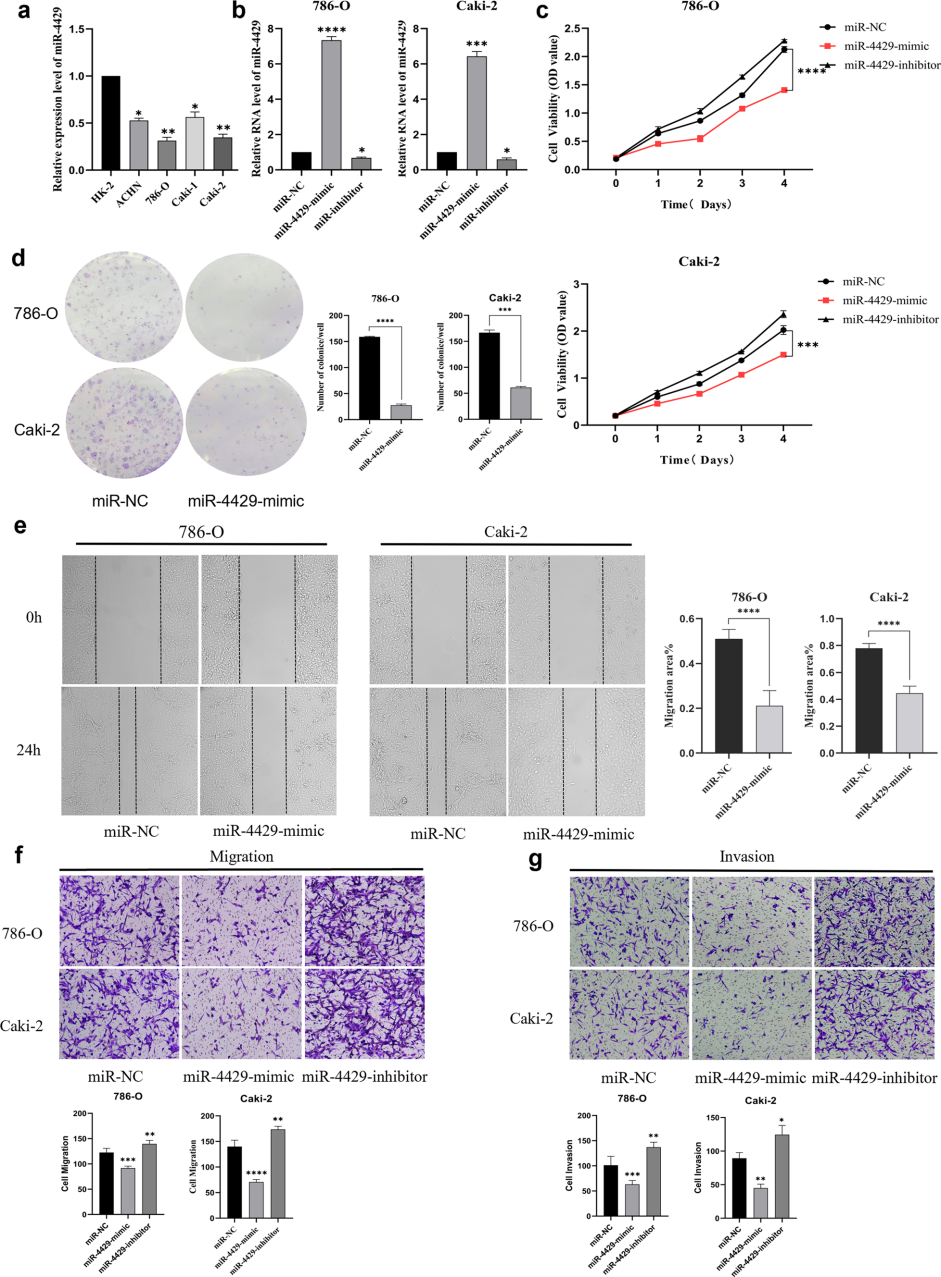
MiR-4429 overexpression inhibited proliferation, migration, and invasion of 786-O and Caki-2 cells in vitro **a** miR-4429 mRNA levels were significantly down-regulated in renal cancer cell lines compared to HK-2 in renal tubular epithelial cells. **b** Transfection of miR-4429-mimic in 786-O and Caki-2 cells significantly up-regulated miR-4429 mRNA levels. **c**–**f** Overexpression of miR-4429 significantly inhibited cell proliferation rate (**c**), clone formation (**d**), wound healing (**e**), migration (**f**), and invasion (**g**). *P < 0.05, **P < 0.01, ***P < 0.001, ****P < *0.0001*

Our findings unveil a substantial downregulation of miR-4429 in 786-O and Caki-2 cell lines (Fig. 2a). Furthermore, the expression level of miR-4429 experienced a significant upregulation following transfection with miR-4429-mimic (Fig. 2b). Importantly, this overexpression of miR-4429 exerted a notable inhibitory effect on cell proliferation (Fig. 2c, d), migration, and invasion (Fig. 2e–g). These compelling results posit miR-4429 as a promising candidate in the role of a tumour suppressor in ccRCC.

### CD274, a potential target of miR-4429, has elevated expression in ccRCC and positively correlates with histologic grading

**Fig. 3:**
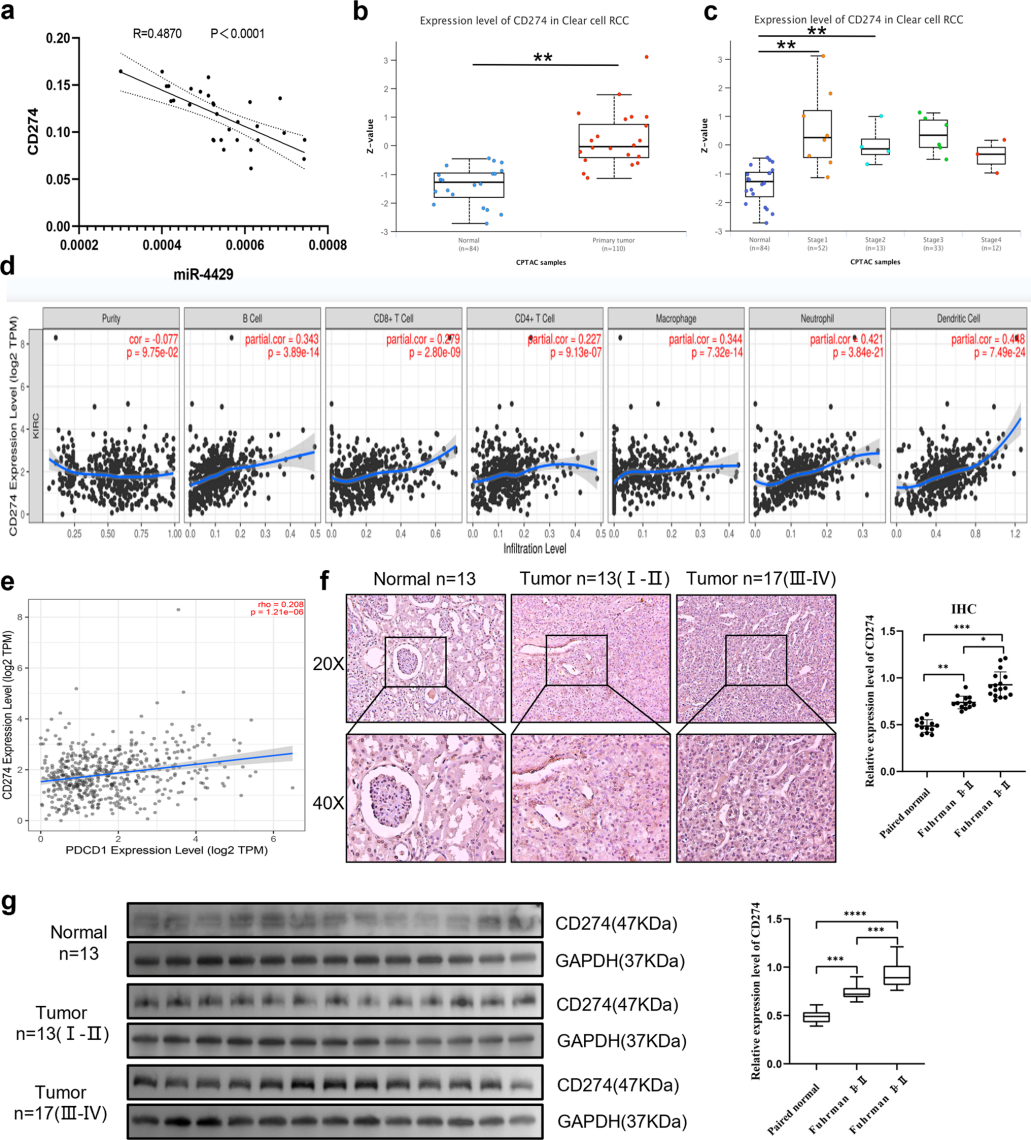
CD274, a potential target of miR-4429, has elevated expression in ccRCC and positively correlates with histologic grading** a** MiR-4429 was negatively correlated with the mRNA level expression of CD274 by qRT-PCR. **b**, **c** The UALCAN database showed that CD274 expression was upregulated in ccRCC and positively correlated with histologic grading. **d** CD274 expression was positively correlated with B cells, CD4^+^ T cells and CD8^+^ T cells, macrophages, neutrophils, and dendritic cell infiltration were positively correlated. **e** The Timer2.0 online database showed a linear positive correlation between PD-1/CD274 expression levels. **f**, **g** IHC and WB assay showed that CD274 expression levels in normal (n = 13), tumor grade I–II (n = 13), and tumor grade III–IV (n = 17) tissues were positively correlated with tumor grade. *P < 0.05, **P < 0.01, ***P < 0.001, ****P < *0.0001*

As widely acknowledged, immune cell infiltration within the tumor microenvironment (TME) can profoundly influence the effectiveness of immunotherapy and the subsequent prognosis of ccRCC patients. Extensive research has demonstrated that CD274 (PD-L1) is prominently overexpressed on the surface of malignant tumour cells [12, 13]. To delve deeper into the potential relationship between miR-4429 and CD274, we evaluated the mRNA expression levels of miR-4429 and CD274 in 30 ccRCC samples using qRT-PCR and subjected them to Pearson correlation analysis. The results unequivocally demonstrated a negative correlation between miR-4429 and CD274 mRNA expression levels (Fig. 3a). In our study, differential expression analysis of the CPTAC protein database within the UALCAN repository revealed an up-regulation of CD274 expression in ccRCC (Fig. 3b). Furthermore, this elevated expression was positively correlated with histological grading (Fig. 3c). Notably, data analysis based on the Timer 2.0 database unveiled a positive association between CD274 expression and the infiltration of B-cells, CD4+ T-cells, CD8+ T-cells, macrophages, neutrophils, and dendritic cells (Fig. 3d). It is well known that CD274 binds to the programmed cell death receptor-1 (PD-1), thereby impeding T cell activation and proliferation, ultimately culminating in immune evasion. Our investigation using the Timer 2.0 online database demonstrated a consistent, linear positive correlation between PD-1/CD274 expression levels (Fig. 3e).

In addition, amongst the tissue samples derived from 30 ccRCC patients. Subsequently, we employed immunohistochemistry(IHC) and Western Blot (WB) to quantify CD274 protein expression levels in Fuhrman I + II (n = 13) and Fuhrman III + IV (n = 17) tumour tissues, as well as their paired normal control tissues (n = 13). Our findings indicated that CD274 protein expression levels were upregulated and positively associated with Fuhrman histological grading when compared to normal tissues (Fig. 3f–g).

The foregoing results underscore the up-regulation of CD274 in ccRCC tumour tissues, its positive correlation with Fuhrman histological grade, and its inverse relationship with miR-4429 expression. This implies that the combination of miR-4429 and CD274 might serve as a potential immunotherapeutic target and prognostic biomarker for patients.

### MiR-4429 regulates its expression and inhibits the PI3K/AKT signaling pathway by targeting CD274 3′-UTR

**Fig. 4:**
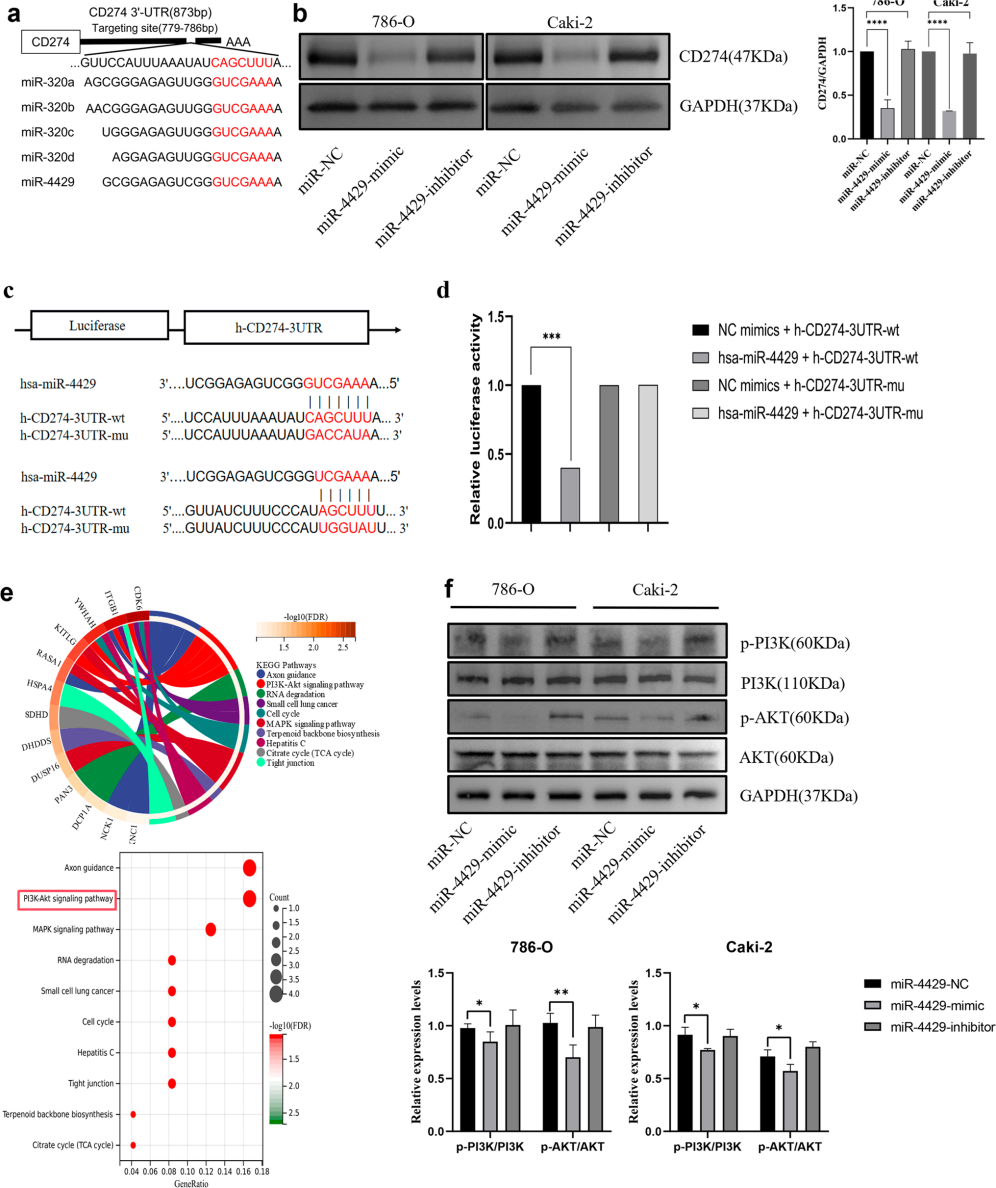
MiR-4429 regulates its expression and inhibits the PI3K/AKT signaling pathway by targeting CD274 3′- UTR **a** predicts potential targets of miR-4429 through miRNA target prediction databases miRDB and TargetScanv7.1. **b** Overexpression of miR-4429 significantly inhibited CD274 expression in ccRCC cell lines 786-O and Caki-2. **c** Dual-luciferase reporter assay was used to verify the targeting of miR-4429 and CD274 and the wild-type and mutant binding sites. **d** MiR-4429 significantly reduced the activity of wild-type double luciferase but had no effect on the mutant. **e** KEGG showed that miR-4429 is associated with signal pathways such as PI3K/AKT and MAPK. **f** Overexpression of miR-4429 significantly inhibited the p-PI3K and p-AKT protein expression, which in turn inhibited PI3K/AKT signaling pathway activation.*P < 0.05, * *P < 0.01, * * *P < 0.001, * *P < *0.0001*

To delve into the potential mechanism underlying miR-4429’s anti-tumour effect, we harnessed miRNA target prediction databases, miRDB and TargetScanv7.1, to predict its potential targets. Our findings revealed that miR-4429 specifically targets the 3′-UTR of CD274 (Fig. 4a). Furthermore, the overexpression of miR-4429 in 786-O and Caki-2 cell lines led to a significant reduction in CD274 expression compared to the miR-NC group (Fig. 4b). The miR-4429’s interaction with CD274 was verified through a dual luciferase assay, with the CD274 wild-type and mutant binding sites clearly delineated in Fig. 4c. Subsequent experiments indicated that miR-4429 significantly reduced the activity of the wild-type dual luciferase, while the mutant showed no discernible effect, conclusively establishing CD274 as a direct target of miR-4429 (Fig. 4d).

Following this, we delved into whether miR-4429 could influence the PI3K/AKT signalling pathway, a pathway intricately linked with CD274 and tumour development [14, 15]. Our study unveiled the KEGG functional enrichment of 1047 potential targets of miR-4429 using the miRDB database. The results unequivocally demonstrated a high correlation between miR-4429's potential target functions and the PI3K/AKT signalling pathway (Fig. 4e). Meanwhile, transfection of miR-4429-mimic into 786-O and Caki-2 cell lines substantially impeded the expression of p-PI3K and p-AKT proteins when compared to the miR-NC group, thereby hampering the activation of the PI3K/AKT signalling pathway (Fig. 4f).

In light of these findings, it is evident that miR-4429 serves to suppress tumour growth in ccRCC by inhibiting the activation of the PI3K/AKT signalling pathway through targeting CD274.

## Discussion

ccRCC presents a challenging clinical scenario characterized by significant immune infiltration. Despite notable advancements in targeted therapies, the prognosis for patients with highly staged and metastatic ccRCC remains grim [16, 17]. Surgical resection stands as the primary treatment for clinically confined ccRCC; however, post-surgical mortality rate remains high, particularly for older patients and those in advanced stages [18]. Adding to the complexity, ccRCC exhibits resistance to chemoradiotherapy and pharmacological interventions, underscoring the pressing need to explore novel molecular targets and biomarkers [19].

MicroRNAs are used as tumor markers in a variety of solid tumors, and there is a strong association with tumorigenesis and progression [20]. MiR-4429, a microRNA positioned on chromosome 2p25.1, has involved in the pathogenesis of various solid tumours [21–24]. For instance, Pan et al. revealed that miR-4429 retards tumour advancement and epithelial-mesenchymal transition by targeting CDK6 in ccRCC [25]. Our investigation has unveiled significant down-regulation of miR-4429 in ccRCC. Moreover, augmenting miR-4429 levels led to a considerable reduction in CD274 expression and subsequent restraint of the PI3K/AKT signalling pathway, signifying the tumour-suppressive potential of miR-4429 in ccRCC through its targeting of CD274 and control over the PI3K/AKT pathway.

Tumour immunogenicity is a contemporary challenge in oncology, with CD274 serving as a focal point for innovative immunotherapeutic strategies [26, 27]. CD274 expression profoundly influences cancer aggressiveness, clinical outcomes, tumour development, and prognosis, establishing itself as an independent adverse prognostic indicator for ccRCC [28, 29]. In our research, we definitively identified CD274 as a direct target of miR-4429 and established a negative correlation between miR-4429 and CD274 mRNA expression levels. Our in vitro experiments demonstrated that miR-4429 overexpression significantly curtails the proliferation, migration, and invasion of ccRCC cells. Additionally, our analysis of the UALCAN database and clinical specimens unveiled the upregulation of CD274 in ccRCC and its positive correlation with the Fuhrman grade and the infiltration of PD-1, Immune infiltrating cells. Hence, the combinatory evaluation of miR-4429 and CD274 bears great potential for assessing the clinical prognosis of ccRCC patients, suggesting a plausible regulatory mechanism for CF274 as an oncogene in ccRCC.

While multiple studies have illuminated the association between abnormal microRNA expression and various intrinsic tumour processes, showcasing the substantial impact of microRNA up- or down-regulation in suppressing tumour cell proliferation, migration, in vitro invasiveness, and subcutaneous tumour growth, the clinical application of miRNA regulation and its targeted therapies remains constrained by limitations. Significantly, ccRCC as an immune-responsive tumour, is likely intertwined with immune system orchestration throughout its development and progression. Hence, modulating the immune response emerges as a promising avenue for ccRCC therapy. Consequently, delving into the potential of the miR-4429/CD274 axis as a target for immunomodulation and immunotherapy in ccRCC is merited. However, further investigations are warranted to ascertain whether CD274 fosters ccRCC progression by sustaining the activation of the PI3K/AKT signalling pathway.

In summary, our study has elucidated that miR-4429 overexpression yields a significant impediment to ccRCC progress, chiefly mediated through the PI3K/AKT signalling pathway. We have pinpointed CD274 as a direct and functional target of miR-4429, with its expression levels correlating with higher histological grading in ccRCC. Therefore, the newfound miR-4429/CD274 axis holds promise as a therapeutic target and prognostic biomarker for ccRCC patients.

### Supplementary Information

Below is the link to the electronic supplementary material

Supplementary file1 (CSV 67 KB)

Supplementary file2 (CSV 40 KB)

Supplementary file3 (CSV 21 KB)

Supplementary file4 (DOCX 15421 KB)

## Data Availability

Data is provided within the manuscript or supplementary information files.
