# Supplementary material for: miR-4429 inhibits ccRCC proliferation, migration, and invasion by directly targeting CD274
Source: Discov Oncol. 2024 May 27;15:190. doi: 10.1007/s12672-024-01055-4 (PMC11130097; doi:10.1007/s12672-024-01055-4)

PI3K P-PI3K


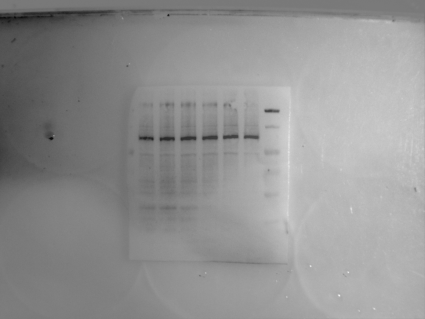

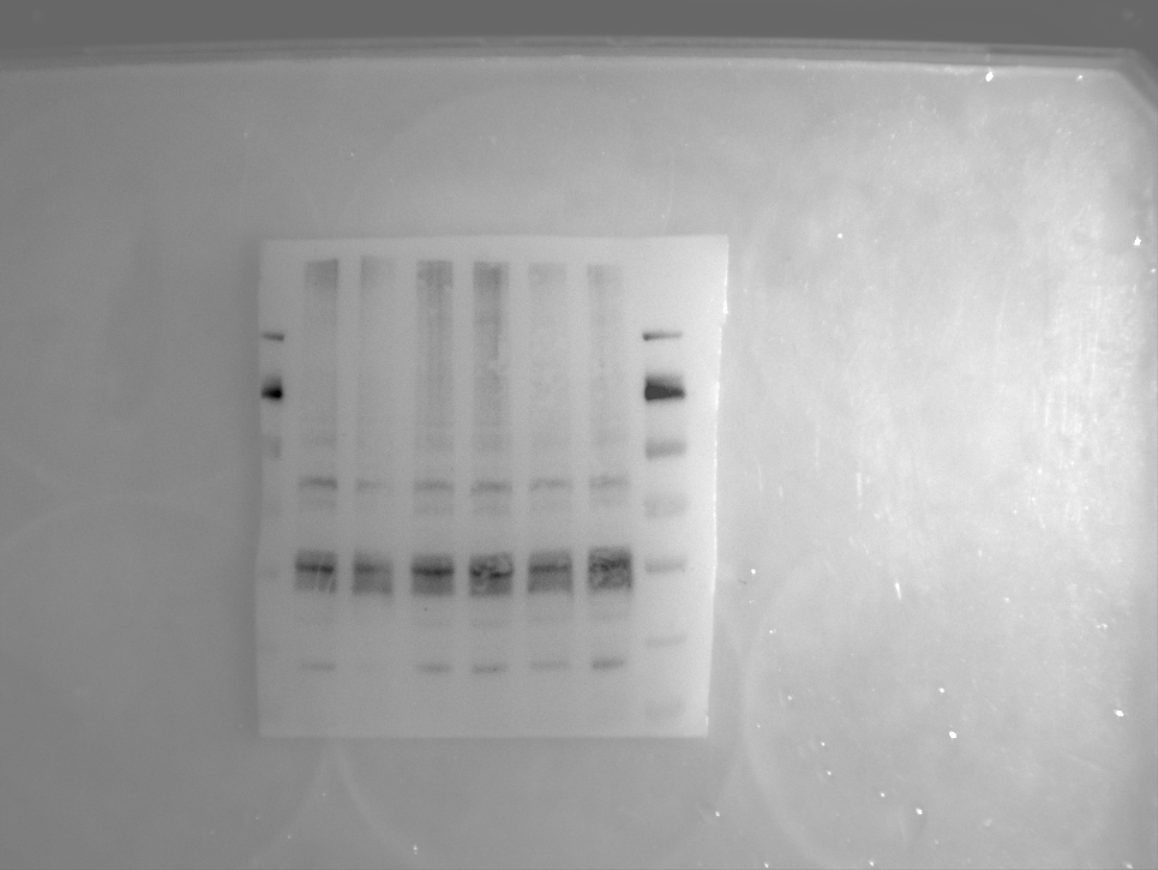


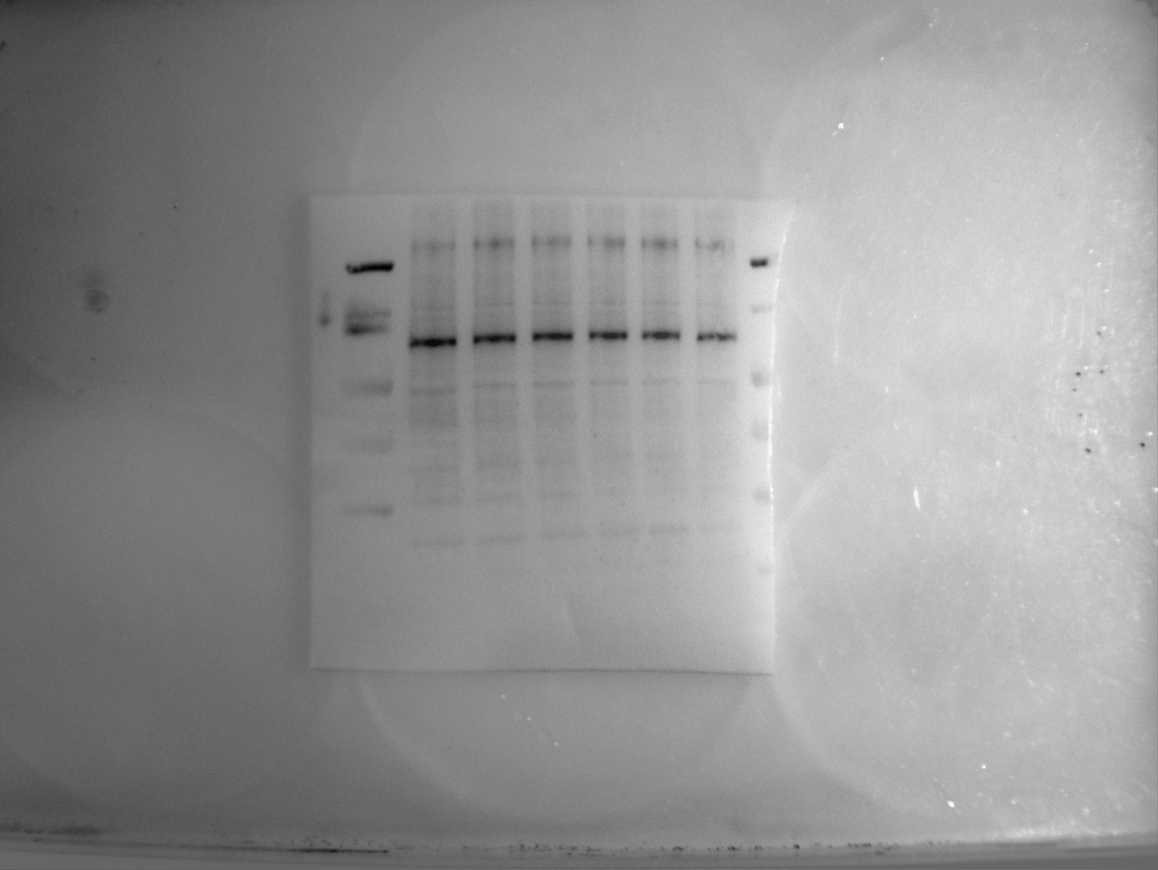

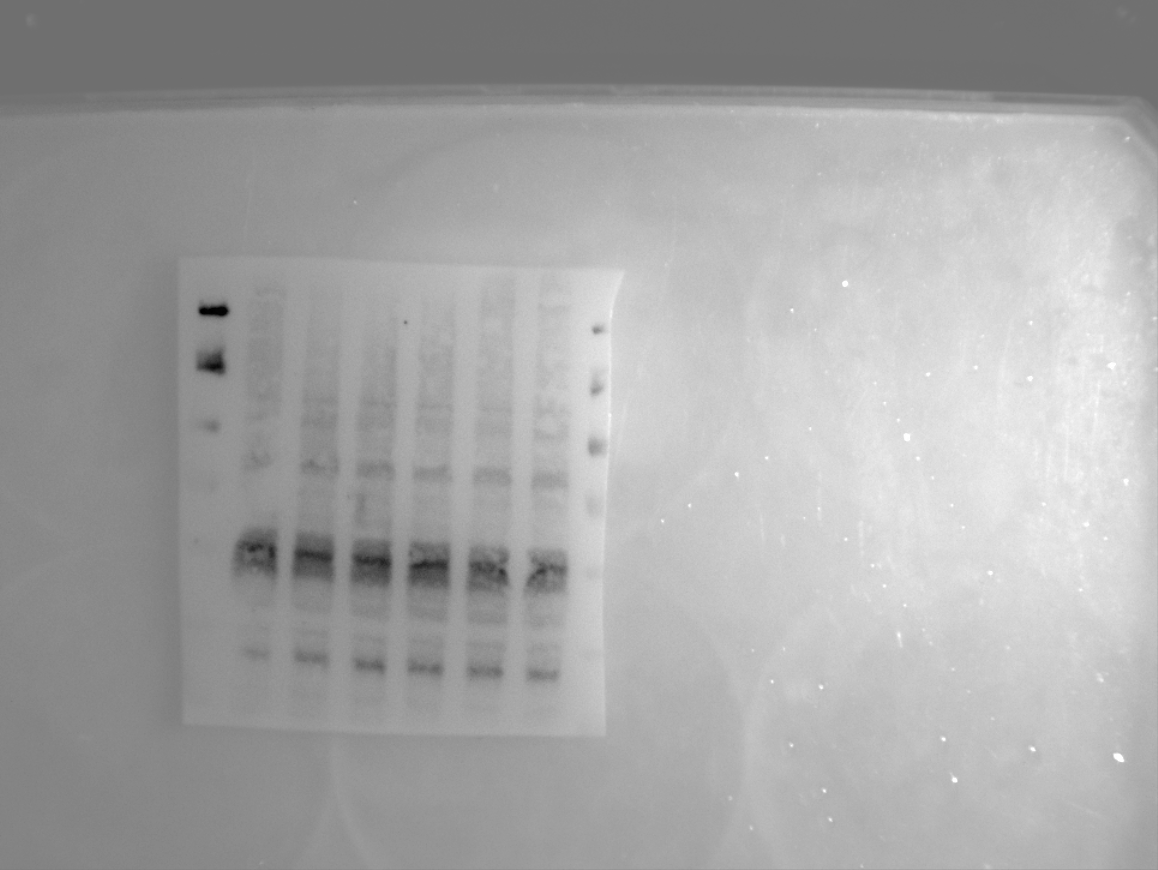


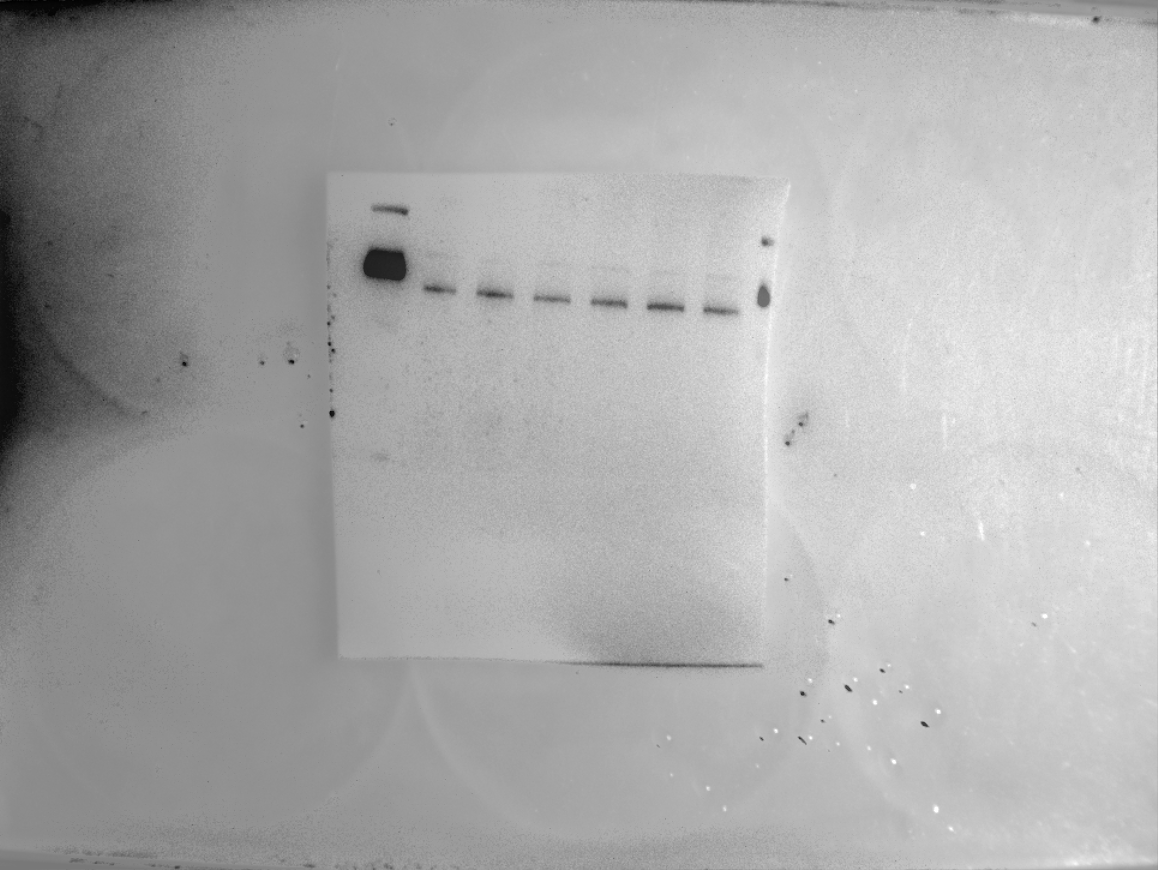

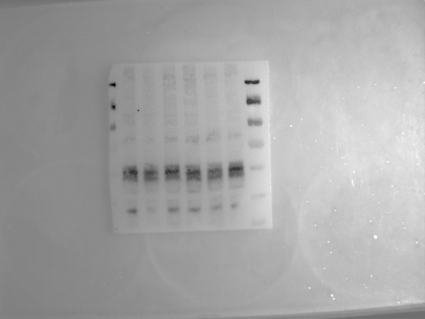


Grouping order Grouping order

786-O Caki-2 786-O Caki-2

NC mimic inhibitor NC mimic inhibitor NC mimic inhibitor NC mimic inhibitor

AKT P-AKT


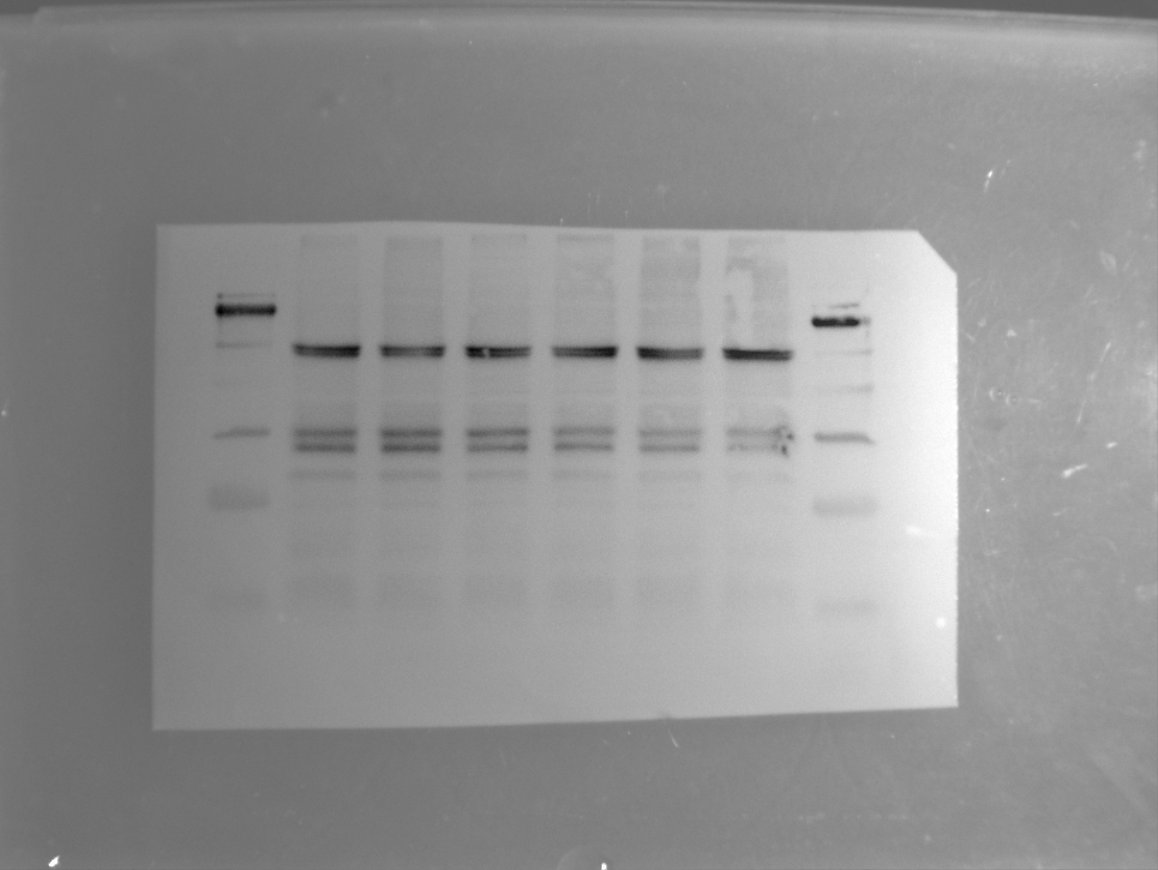

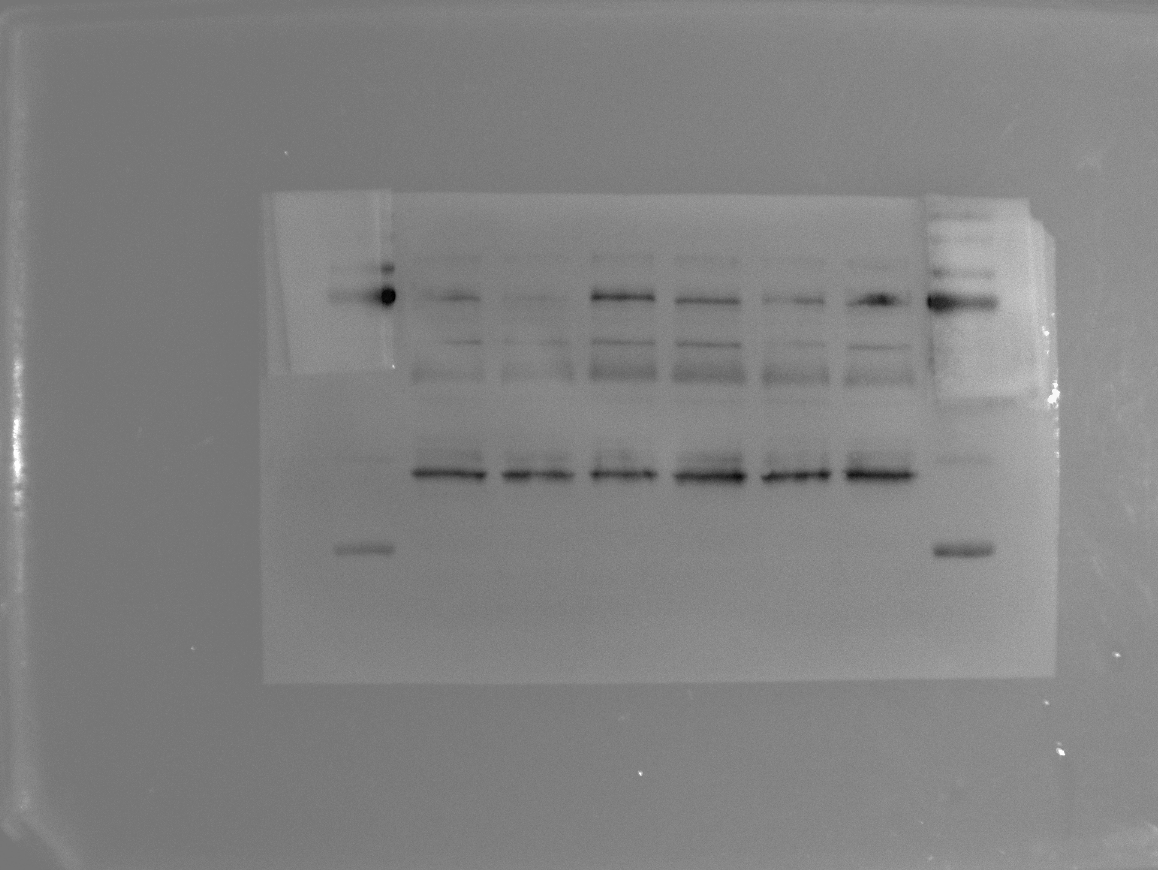


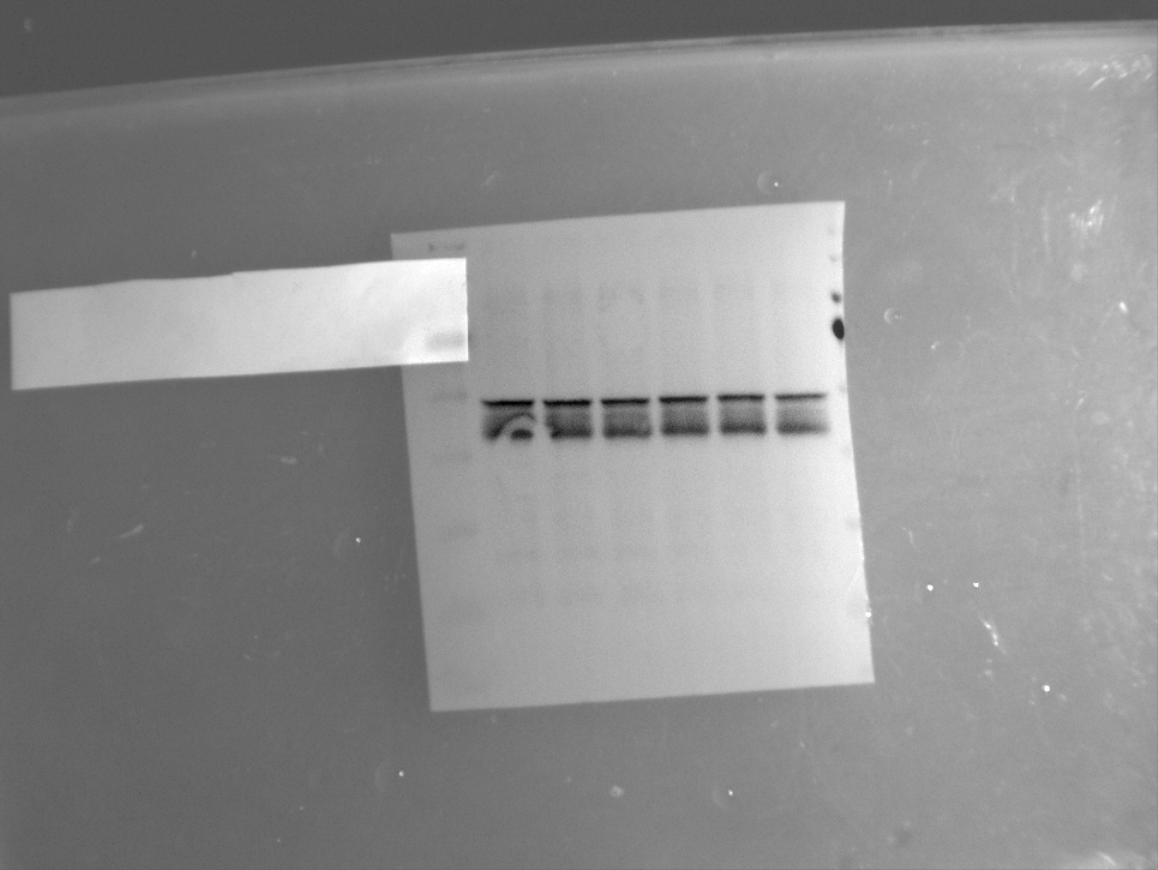

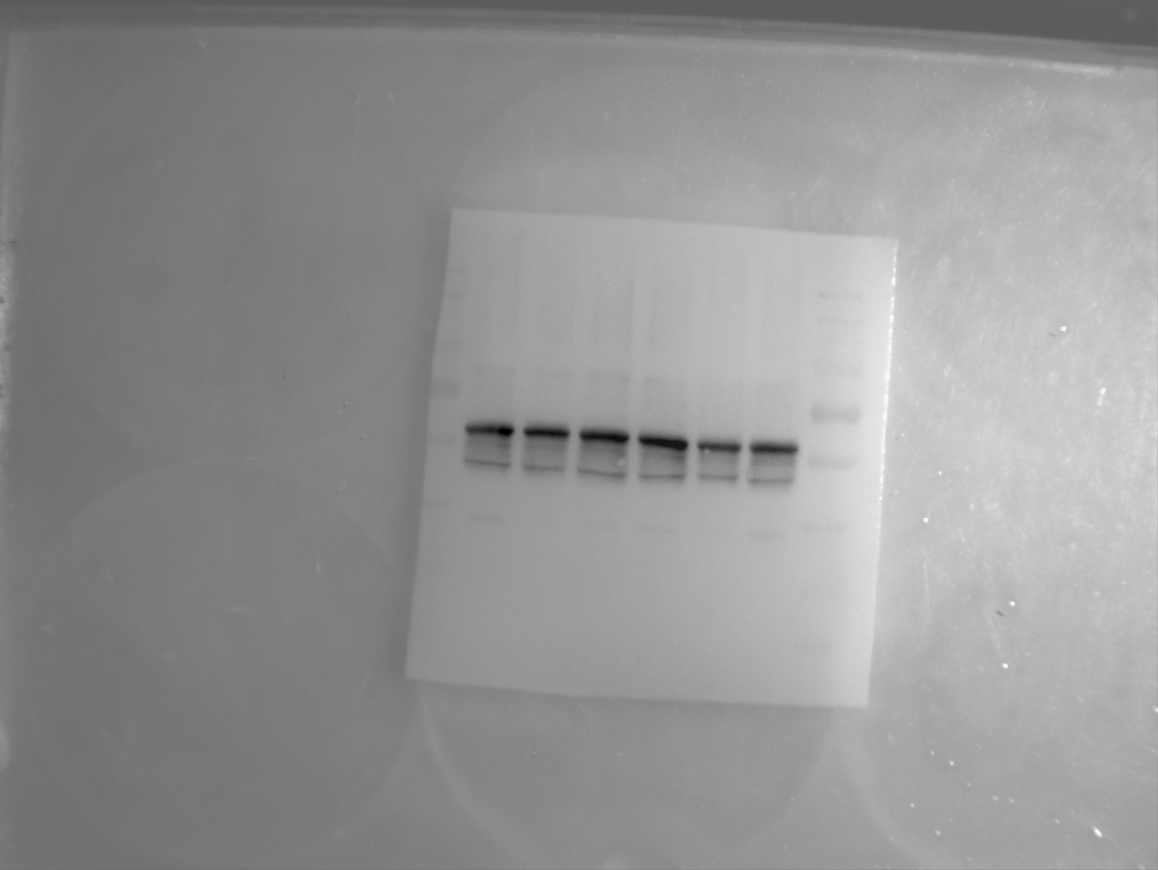


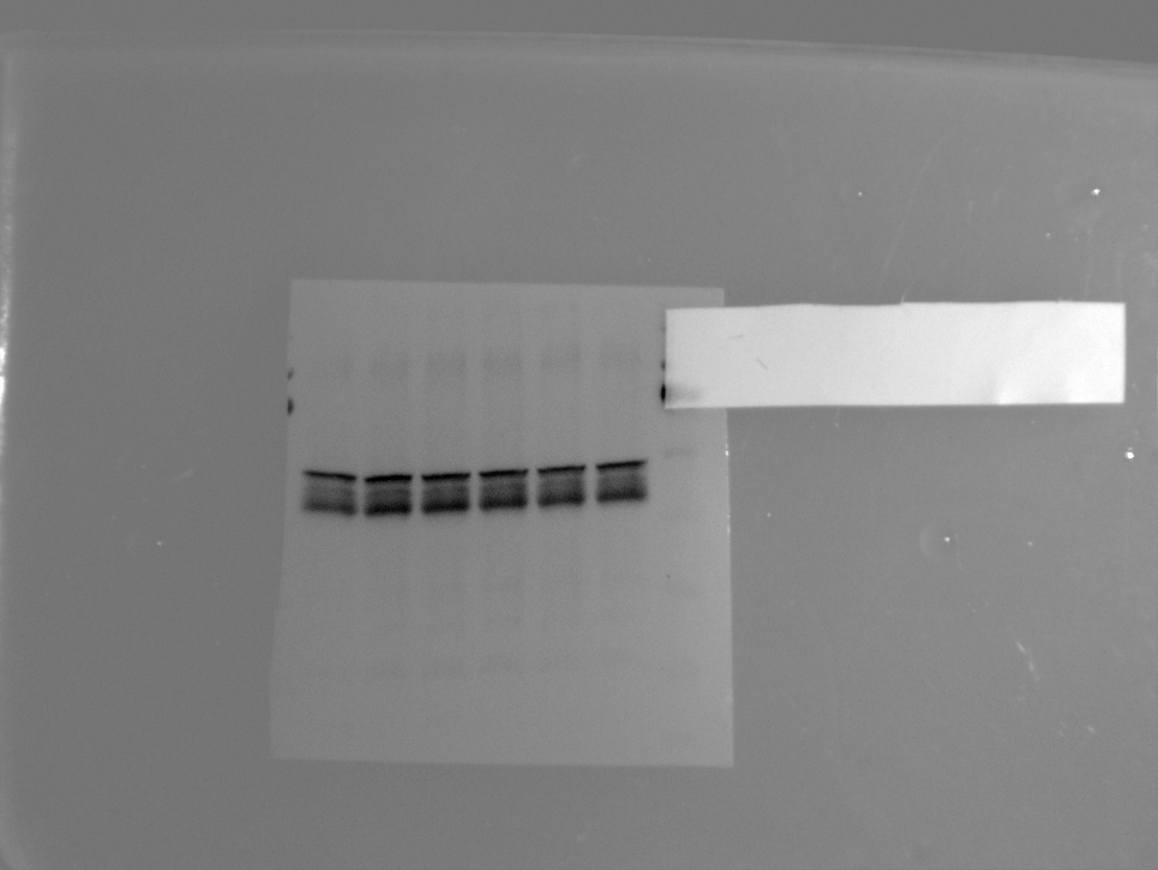

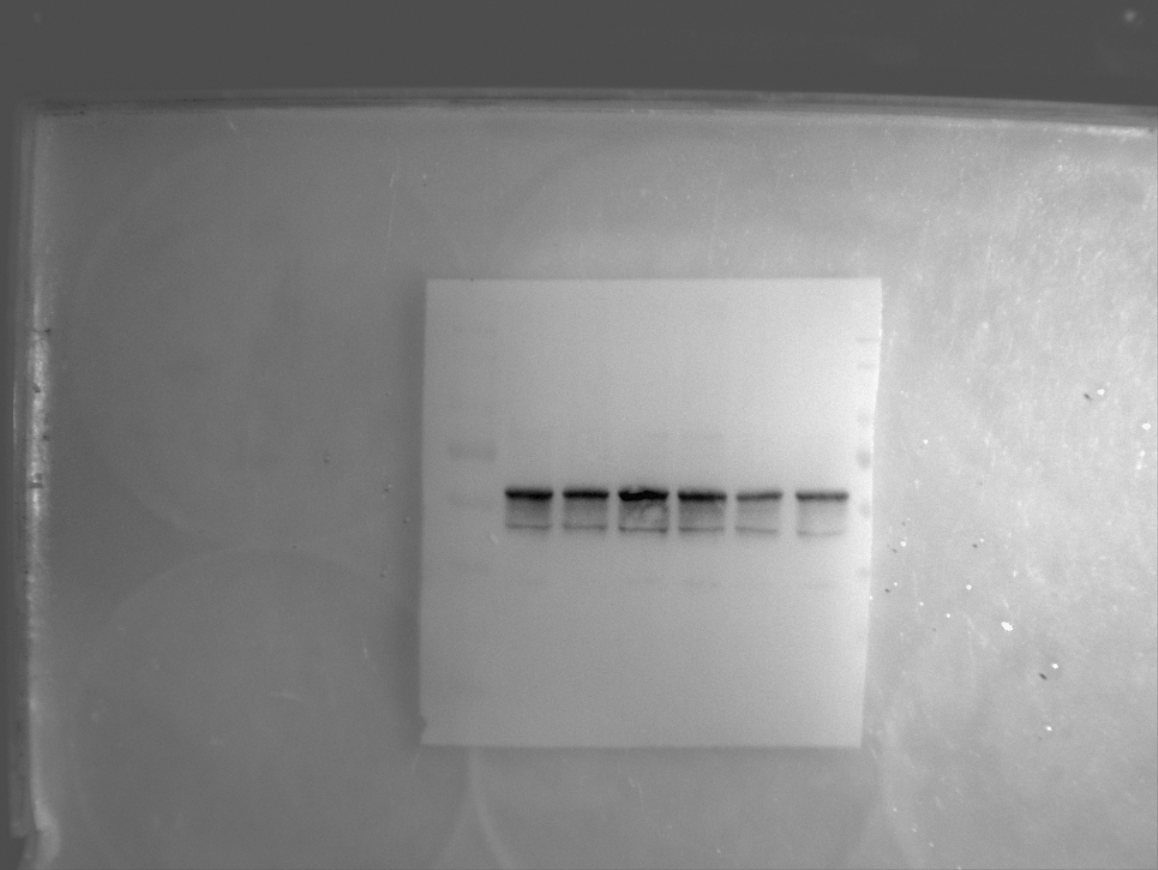


Grouping order Grouping order

786-O Caki-2 786-O Caki-2

NC mimic inhibitor NC mimic inhibitor NC mimic inhibitor NC mimic inhibitor

PD-L1 GAPDH


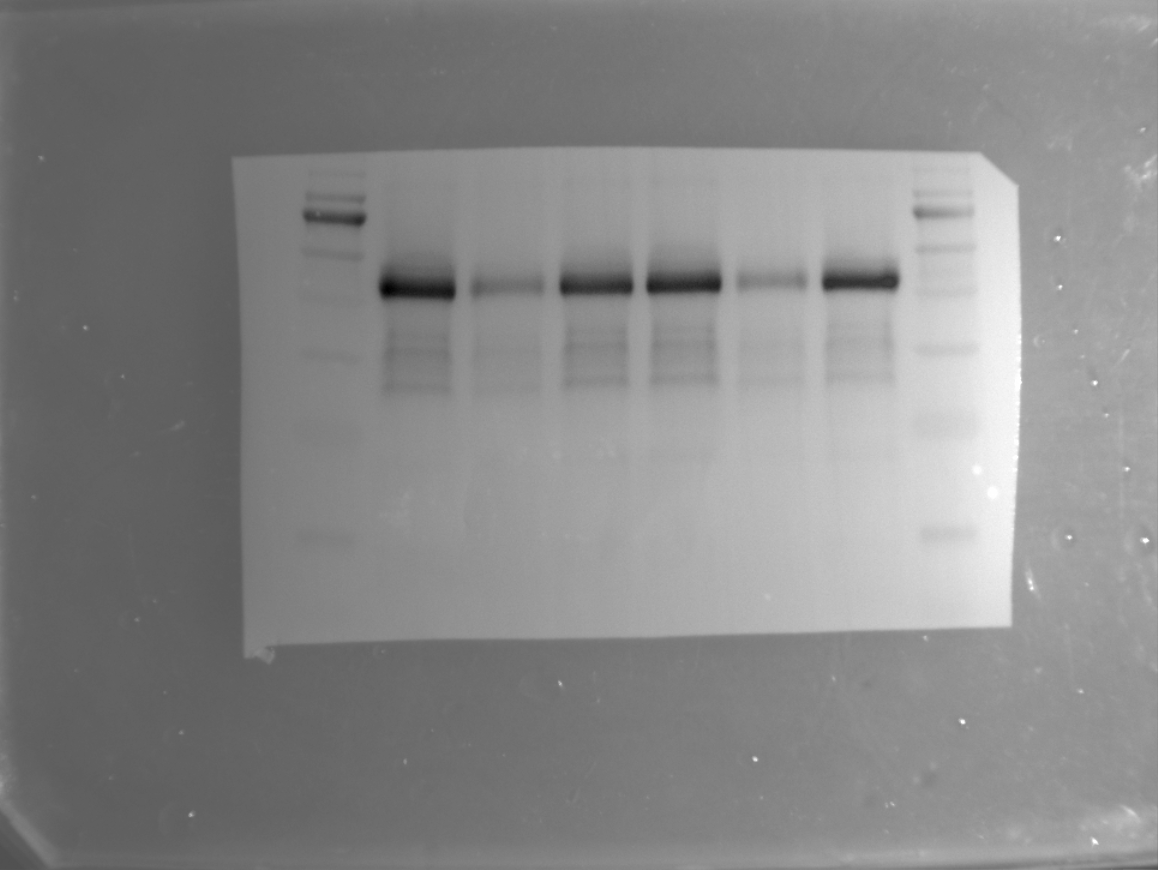

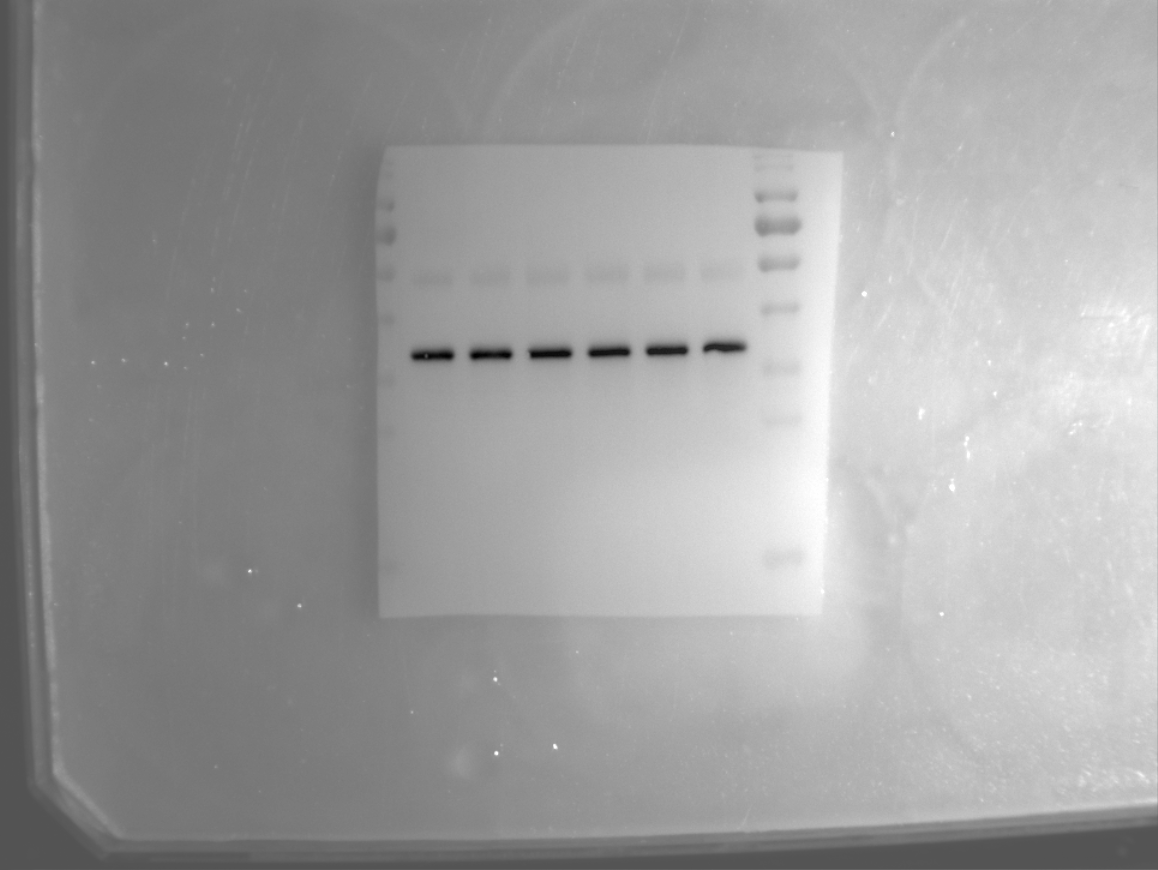


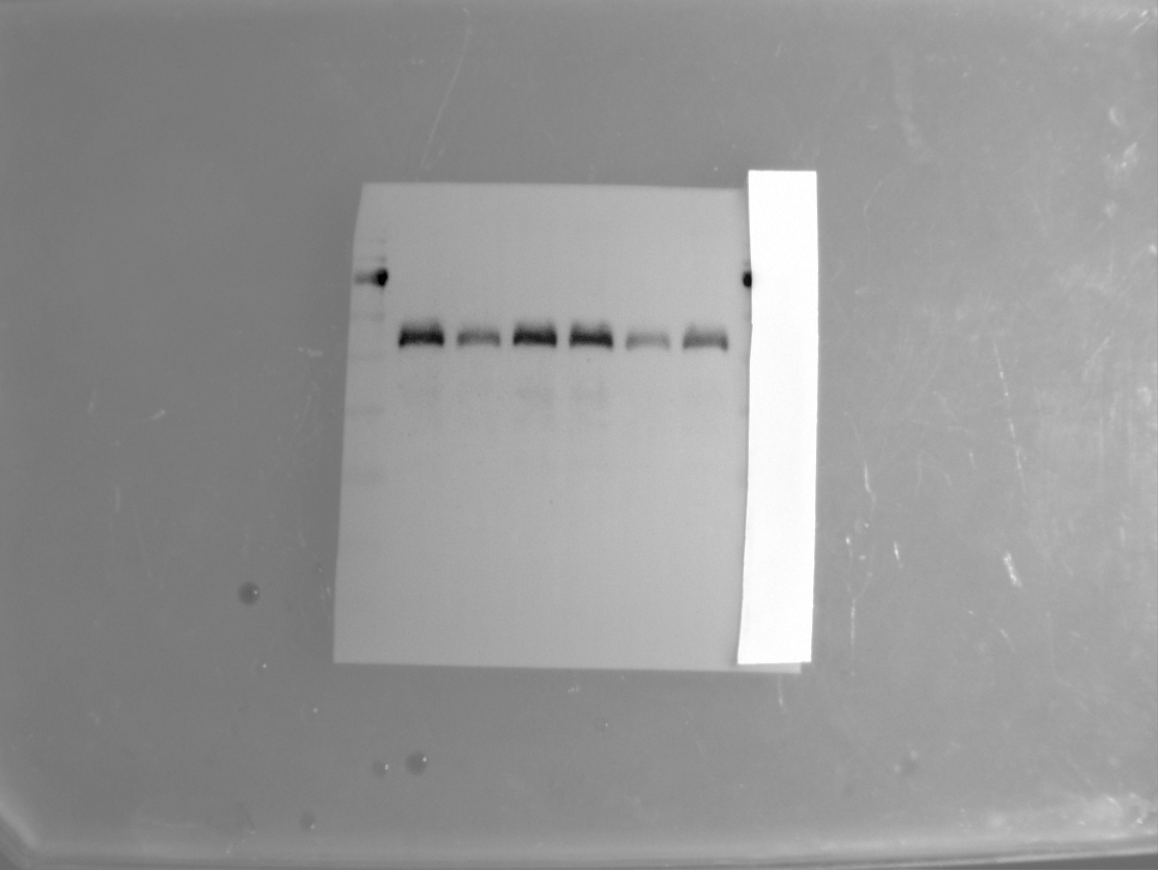

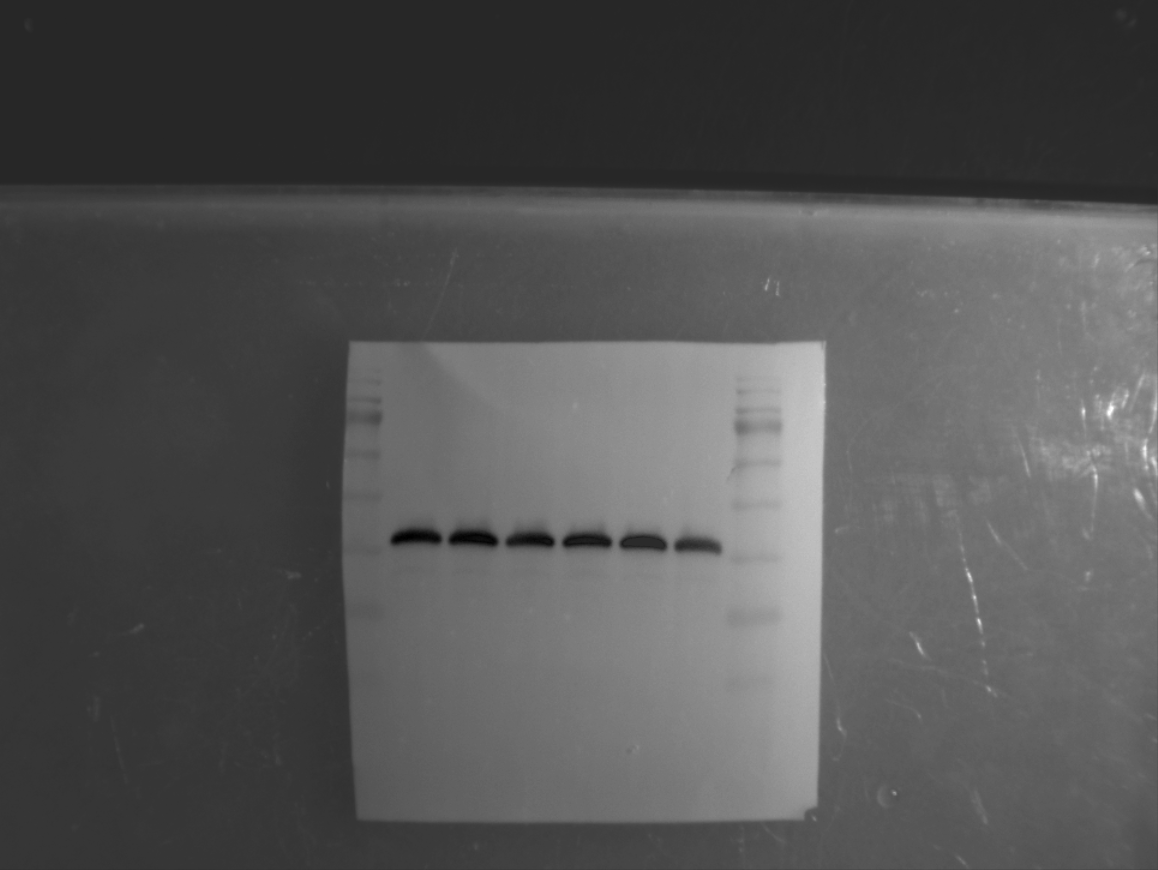


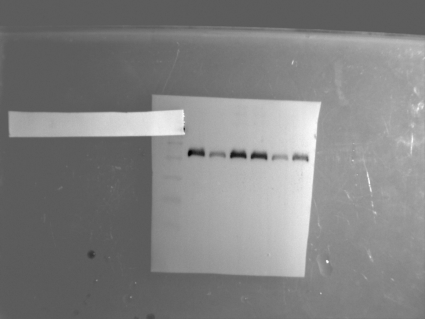

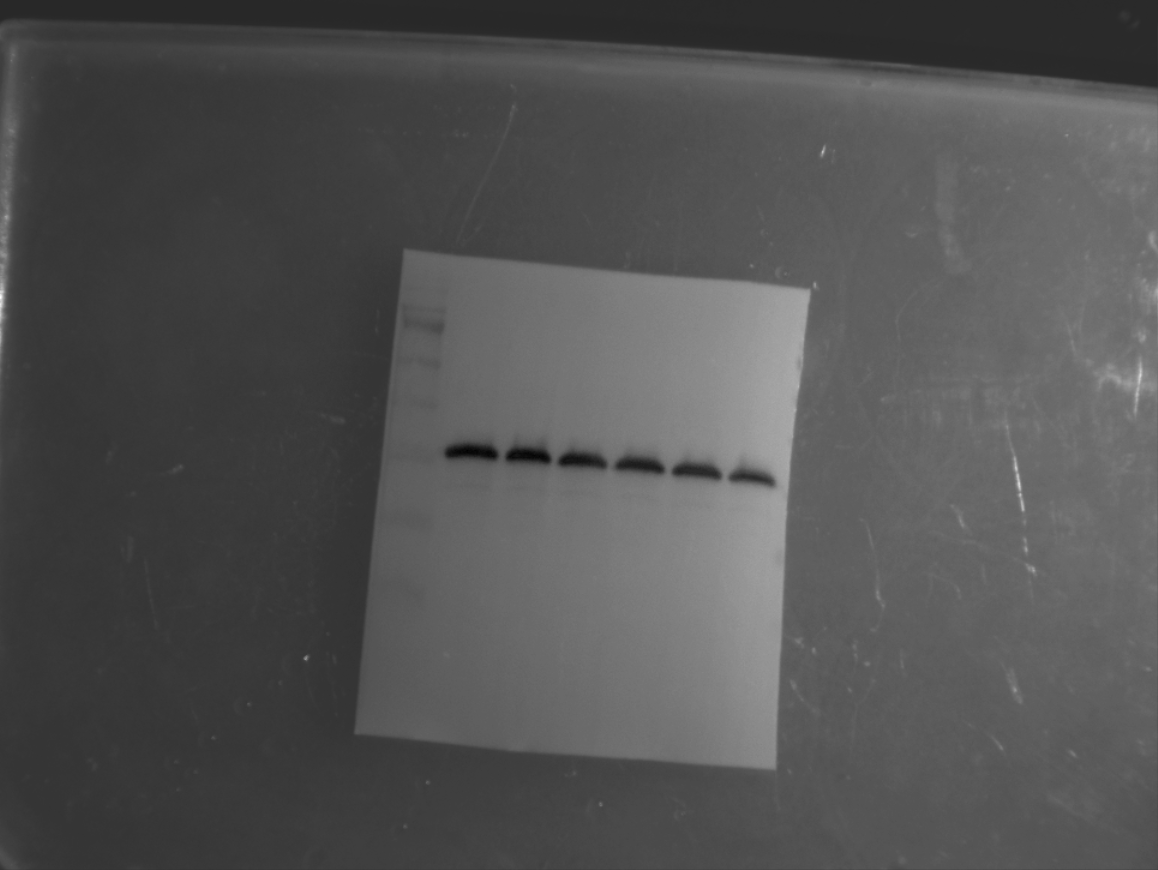


Grouping order Grouping order

786-O Caki-2 786-O Caki-2

NC mimic inhibitor NC mimic inhibitor NC mimic inhibitor NC mimic inhibitor

CcRCC Tissue PD-L1

Furhman I + II (n = 13) Furhman Ⅲ + Ⅳ (n = 18)


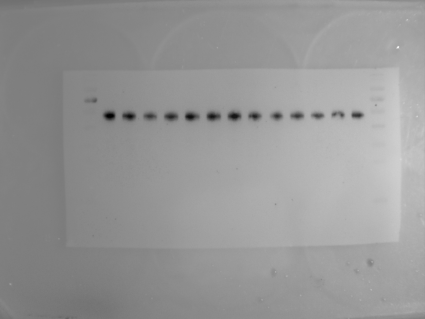

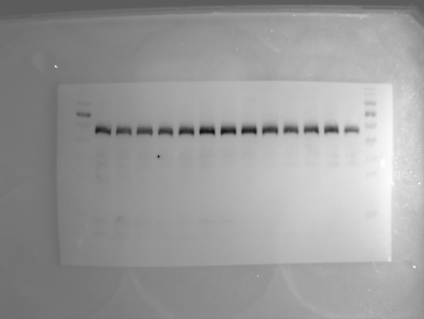


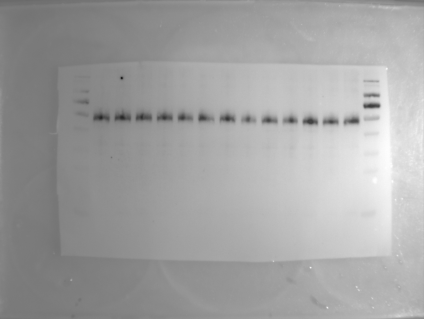

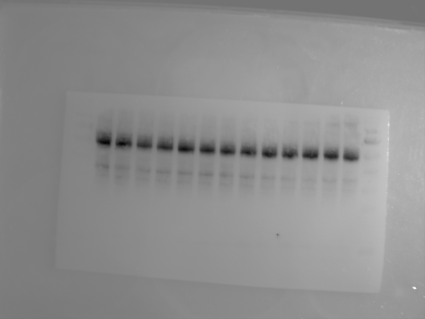


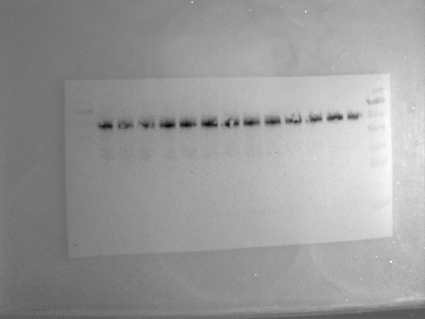

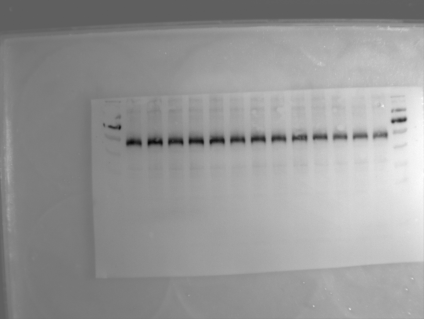


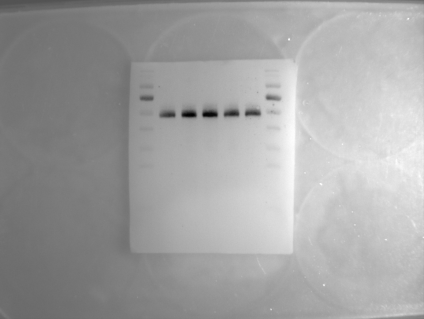


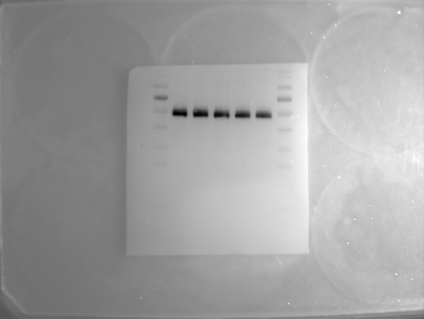


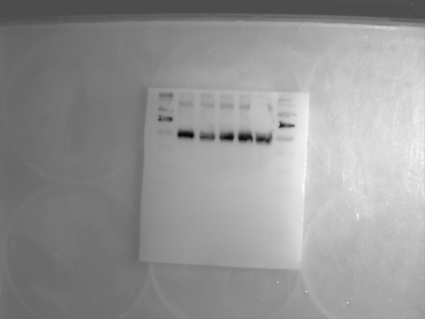


CcRCC Tissue GAPDH

Furhman I + II (n = 13) Furhman Ⅲ + Ⅳ (n = 18)


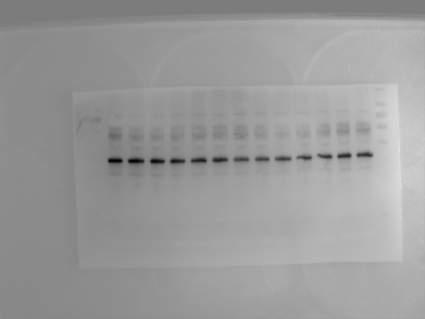

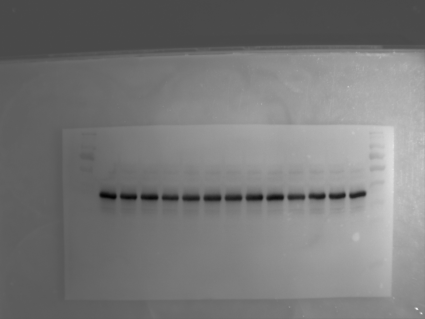


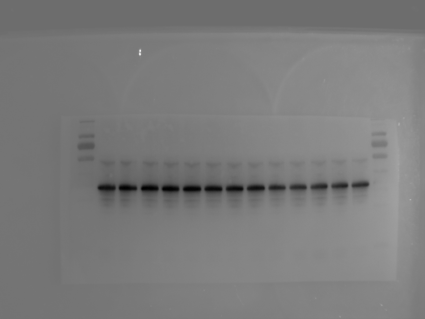

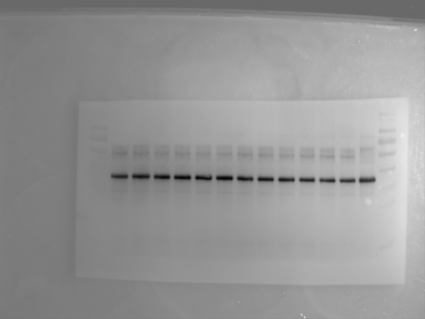


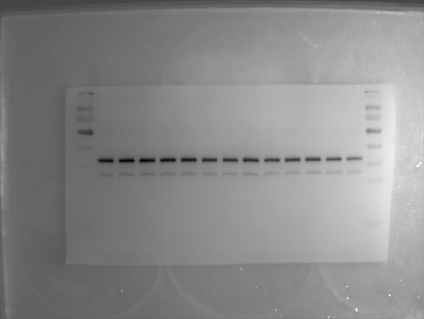

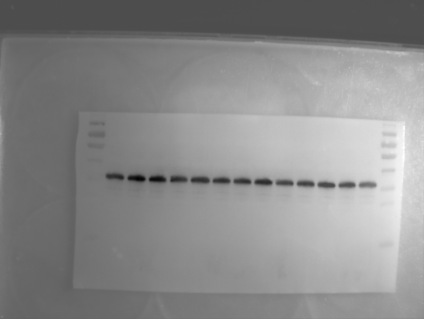


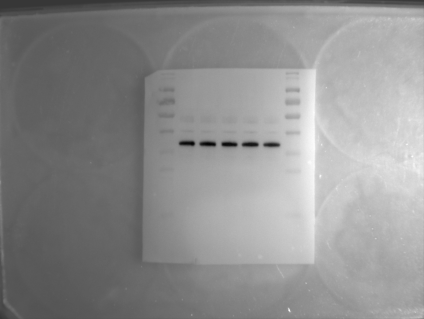


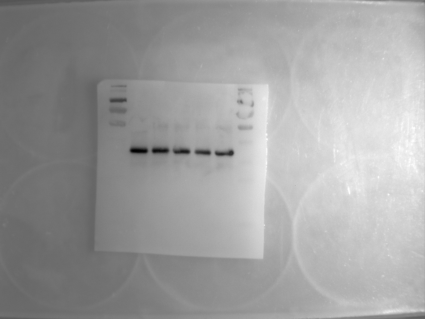


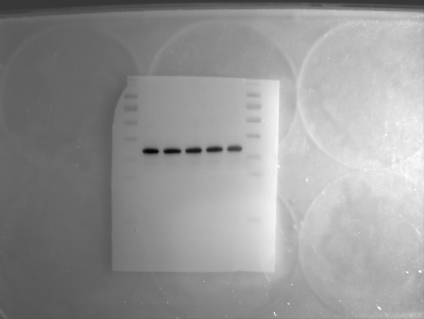


Normal tissue PD-L1 Normal tissue GAPDH

n = 13 n = 13


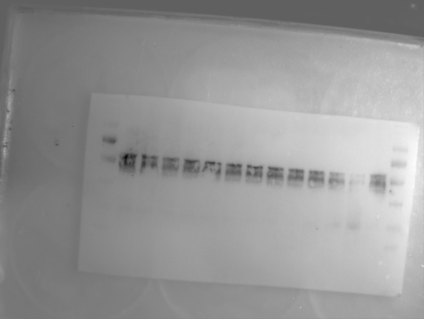

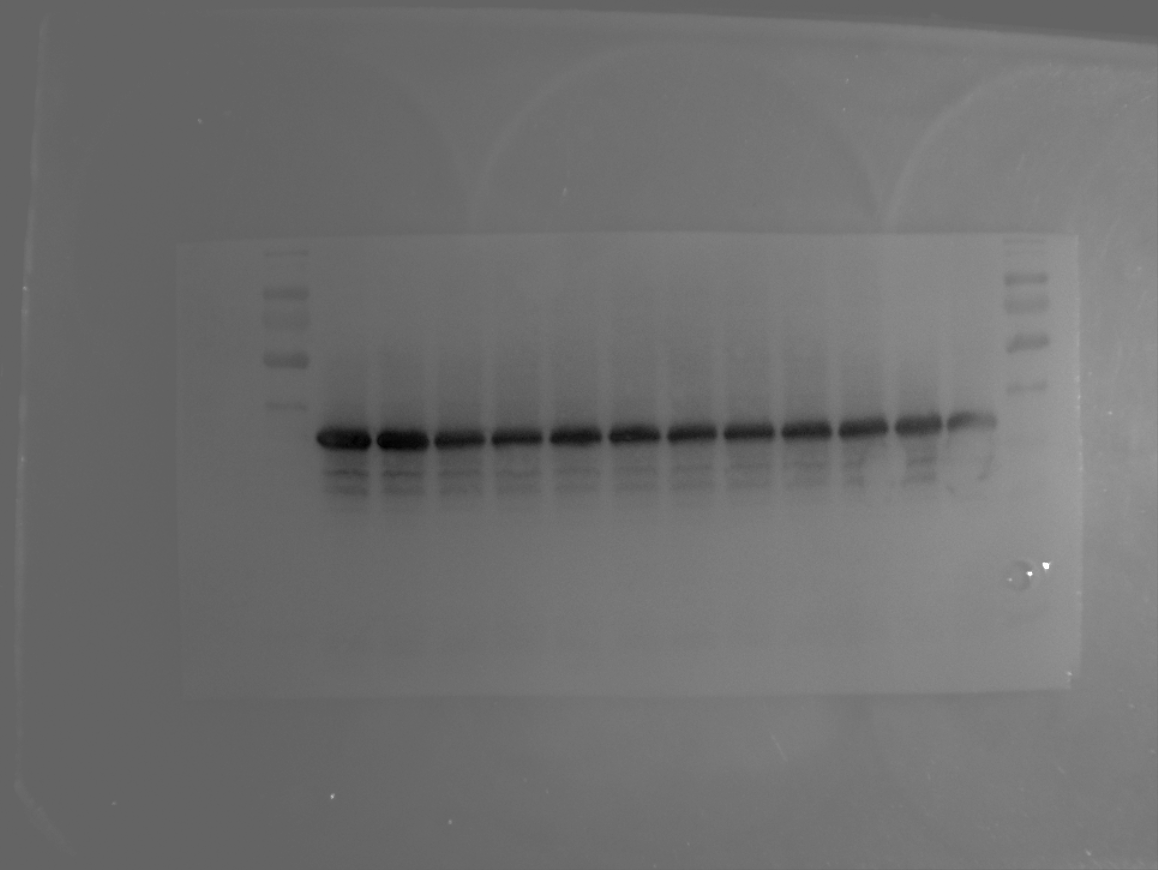


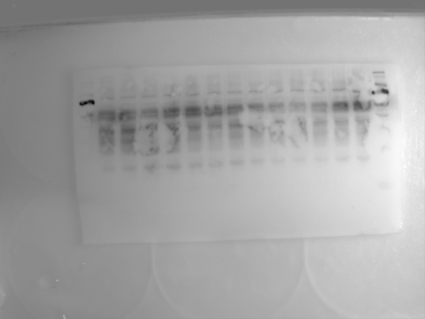

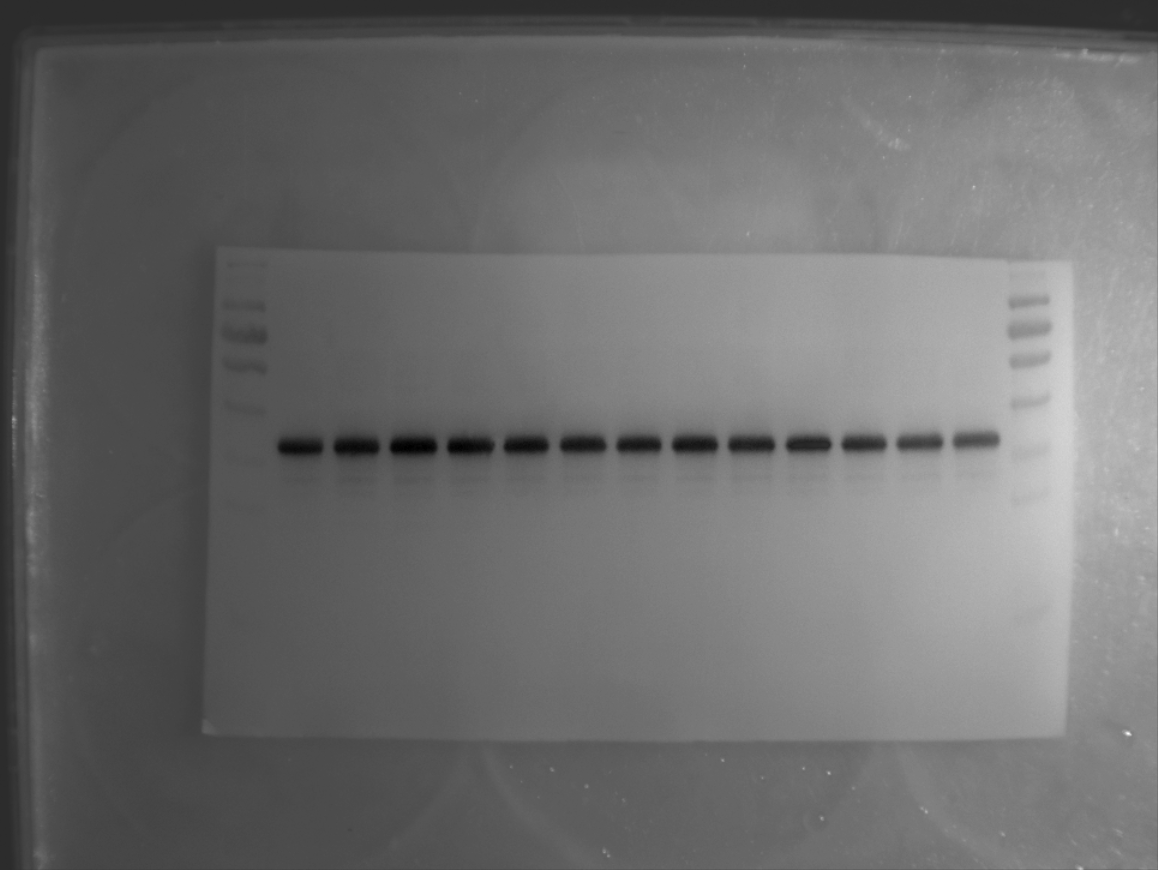


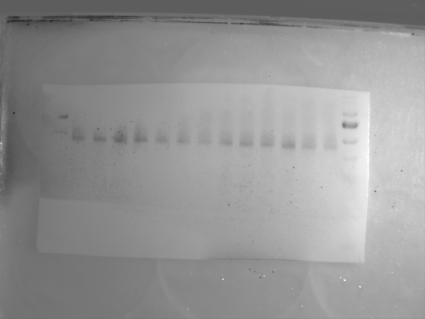

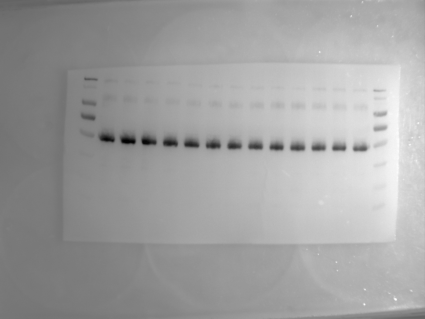

Supplement: Supplementary file 4 — Supplementary file4 (DOCX 15421 KB) [file 12672_2024_1055_MOESM4_ESM.docx]
